# Supplementary material for: “...In Kiambu county, there are different things being done”: A qualitative exploration of healthcare workers’ experiences with cord care practices
Source: PLoS One. 2025 Jun 18;20(6):e0326506. doi: 10.1371/journal.pone.0326506 (PMC12176292; doi:10.1371/journal.pone.0326506)
Supplement: S1 File — This document contains the transcriptions of the IDIs and the indices used for each participant. (PDF) [file pone.0326506.s001.pdf]

INDEX 23 – NAZARETH HOSPITAL

QUALITATIVE STUDY

A SEMI-STRUCTURED QUESTIONNAIRE

This questionnaire will be used to guide the interviewer.

Please let us record the following information that will be held confidentially and used only for the purpose of this research.

Bio data of the interviewee

Age…………………………… Professional line…………………….

Years of practice ……………..

Period of stay (years) in the specific station ……………………………

1. Please describe the existing cord care practices in our Kiambu County

The existing cord care practices. For facility, we use surgical spirit. Because we realized the use of chlorhexidine, majority of the babies would come with NNS. So what we do, we ensure the mother before discarge they have the surgical spirit and they are taught on cord care, demonstrated

2. How would you describe your experience with CHX cord care antiseptic use in your hospital?

In the facility, we don't use chlorhexidine, as I said earlier. We use surgical spirit, first of all, because they're not available, they're not delivered by KEMSA. A bottle of chlorhexidine is around 200. So a lot of mothers are not able to afford compared to surgical spirit, which is around 50. Then as I said, chlorhexidine has higher cases of NNS, especially the cream. Yeah.

3. **Kindly describe the factors facilitating provision of chlorhexidine gel/solution**

On the case of chlorhexidine, I would not say that we have factors facilitating the provision because we haven't had. We have never had a supply of chlorhexidine. But if asked to prefer the solution to the gel, then it'll be a bit cheaper, maybe 50 shillings that the mothers, if we ask them to buy, that they're able to afford.

4. **What Challenges do you experience with provision of chlorhexidine gel?**

The challenges of provision of chlorhexidine gel, first of all, I'd say it's not readily available. As in, even if I sent a mother outside here to buy, I don't think it's as available as there rest. Secondly, the cost. It's a challenge because the mothers are not able to afford it. Thirdly, the effectiveness, either due to the condition. When it's the gel and is supposed to apply and the mother's hygiene is not very good, the outcome will essentially not be good. And if the outcome is not good, we will not keep prescribing the same thing for the patients. Because it has to be something that works. So I think for the challenges, if they could consider maybe, I think the drop-like. The gel would be less favorable than the drop. In essence that assume this mother [inaudible] mother, how many times do they bath this newborn? Because if they don't and continue applying the gel on a dirty, it won't work. So I would say cost, availability, and the effectiveness.

5. **What would you suggest would lead to an increase in use of CHX?**

I would suggest the government to do more research on the effectiveness of Hexichlor before making it as a policy for use.

6. **Comment on the IEC materials on CHX**

On the issue of IEC materials, they're not available in our facility. If they can be effective and brought down to the facilities, I would recommend they be more pictorial. By a glance, someone is able to know what it is and how it can be used and the readings big enough to be legible. Yes.

INDEX 23 – NAZARETH HOSPITAL

QUALITATIVE STUDY

A SEMI-STRUCTURED QUESTIONNAIRE

This questionnaire will be used to guide the interviewer.

Please let us record the following information that will be held confidentially and used only for the purpose of this research.

Bio data of the interviewee

Age…………………………… Professional line…………………….

Years of practice ……………..

Period of stay (years) in the specific station ……………………………

1. Please describe the existing cord care practices in our Kiambu County

Okay. Right from when the baby is delivered and when the cord is clamped, we normally educate the mothers on the cord care, which includes like wiping the cord with surgical spirit, yeah, to avoid infection.

2. How would you describe your experience with CHX cord care antiseptic use in your hospital?

Okay. The experience with chlorhexidine is that a person use it. But for the patient, most of them are not keen, especially with the tube one that's creamish. They don't wipe it. So most of them come back with a cord infection. Yeah.

Interviewer: That is for the gel?

Interviewee: *Yeah, for the gel. Yeah.*

Interviewer: *Any-*

Interviewee: *But for the liquid one, it's okay.*

3. Kindly describe the factors facilitating provision of chlorhexidine gel/solution

For the chlorhexidine solution and the gel at the facility, it's not available. Yeah.

4. What Challenges do you experience with provision of chlorhexidine gel?

Okay. The factors are, number one, the unavailability of the chlorhexidine, which I think if it's well marketed in the facilities and the patients are taught about it or its whereabout to facilitates its availability and use in the facility by the patients.

5. What would you suggest would lead to an increase in use of CHX?

So the factors would be, number one, the healthcare providers are given, what's it called? They're taught about the chlorhexidine, the usage, and how to teach the mothers on the usage, and again, the availability of the chlorhexidine in the facilities. Yeah. Yeah.

Interviewer: *Have you ever been taught on the use of chlorhexidine?*

Interviewee: *No.*

6. Comment on the IEC materials on CHX

I've seen the IEC in Kenyatta National Hospital in the labor ward and postnatal. But in the facility in Githunguri, I've not seen any. And I think CME is about the chlorhexidine would really help in creating awareness of the... Is it the drug? Yeah.everywhere, in every ward, in the postnatal rooms, and in labor ward, and also here in antenatal.

INDEX 23 – NAZARETH HOSPITAL

QUALITATIVE STUDY

A SEMI-STRUCTURED QUESTIONNAIRE

This questionnaire will be used to guide the interviewer.

Please let us record the following information that will be held confidentially and used only for the purpose of this research.

Bio data of the interviewee

Age…………………………… Professional line…………………….

Years of practice ……………..

Period of stay (years) in the specific station ……………………………

1. Please describe the existing cord care practices in our Kiambu County

Previously, we used Hexichlor, but for those mothers who were using Hexichlor, they used to come back with the kids with sepsis because it's somehow solid. So most women, they don't know how to apply it. But we introduced surgical spirit, and it worked because most mothers who were using surgical spirits, the cord used to be... It was easy for them to use it because its somehow light, it's watery. So when they're cleaning the cord, it used to help them. So most cords which used to use surgical spirit, they healed very fast than the one which used Hexichlor.

2. How would you describe your experience with CHX cord care antiseptic use in your hospital?

So chlorhexidine now, not Hexichlor?

Interviewer: *It's the same.*

Interviewee: *Hexichlor is-*

Speaker 3: *Yeah. We got the cream before, then we were given the [inaudible].*

Interviewee: *We used to get the supplies of chlorhexidine, but it's no longer... We no longer receive it. We no longer use it.*

Interviewer: *You're no longer using it?*

Interviewee: *Yes.*

3. Kindly describe the factors facilitating provision of chlorhexidine gel/solution

It's because of maybe the outcome, the end results. Maybe they found out that it was not working well. I cannot justify about the finances because I don't know because I... Mostly ni finances and with the supplies.

4. What Challenges do you experience with provision of chlorhexidine gel?

The challenges we have with the provision of supplier of chlorhexidine, it's because it's not on the supply. It's not continuous. It comes for a short while, and it's out of stock.

Interviewer: *When is there, do people like it? Are they using it?*

Interviewee: *When we have the... How can we call... When we have the liquid or the whatever, it's so easy to use. But when it's that solid thing.*

Interviewer: *Gel.*

Interviewee: *The gel, whatever, the mothers gets hard to use it. Some don't know how to apply it. Others apply without cleaning the cord. That's why it was really a challenge to discharge from this mother will go gel.*

Interviewer: *What are the alternative when you don't have any, when you don't have both?*

Interviewee: *When we don't have chlorhexidine liquid and gel. They use surgical spirit.*

Interviewer: *Given the three of them, what would you choose to use?*

Interviewee: Well, there's a method when you don't have any, and you have seen financial status cannot buy if you prescribed. We just tell them to wash the cord with clean water, lukewarm and a clean towel at home. It also works.

Interviewer: Thank you.

5. What would you suggest would lead to an increase in use of CHX?

W suggest its sensitization and the mother needs the education on how to use it because whichever end results, it will determine of the mother use. If they get educated, sensitized, it can be well with them. It can work well.

Interviewer: Are you trained in it?

Interviewee: Trained about?

Interviewer: Use of chlorhexidine.

Interviewee: Chlorhexidine. We were trained when it came first.

Interviewer: Okay.

6. Comment on the IEC materials on CHX

Ward. both in labor ward and postnatal ward.

Interviewer: What would you comment about them.

Interviewee: They're really educative.

Interviewer: Are they available?

Interviewee: As you are explaining to the mother one how to use it, at least the procedures there on how it's being done,

Interviewer: The ones you have, do they have adequate information?

Interviewee: It's adequate.

Interviewer: Okay. And they're available in every area that you'd want them to be?

Interviewee: No. For now, they are few because you can see [inaudible]. It's not there. They're not adequate.

Interviewer: *Okay. Thank you.*

INDEX 23 – NAZARETH HOSPITAL

QUALITATIVE STUDY

A SEMI-STRUCTURED QUESTIONNAIRE

This questionnaire will be used to guide the interviewer.

Please let us record the following information that will be held confidentially and used only for the purpose of this research.

Bio data of the interviewee

Age…………………………… Professional line…………………….

Years of practice ……………..

Period of stay (years) in the specific station ……………………………..

1. Please describe the existing cord care practices in our Kiambu County

So generally for cord care, what I've noted in the facilities that I've practiced, I've practiced in Thika Level 5 and Gatundu Level 5 and also in Kihara Level 4 currently. And what I've noted, there is less uptake of chlorhexidine use for cord care compared to the surgical spirit. And I think the largest contributor of the slow uptake of the chlorhexidine is the skin reaction that comes with it. And also, I think the knowledge gap on how to use it, avoiding the other areas of the skin. So what I've seen, most of the practitioners, the pediatrician and also other practitioners prefer, I've noted the persistent on the use of surgical spirit as opposed to chlorhexidine. But by and large, from what has been there in the literature and what has been there in the current whatever, chlorhexidine has shown well used has a positive outcome in cord care and also preventing related umbilical infection.

2. How would you describe your experience with CHX cord care antiseptic use in your hospital?

So from my personal experience of the use of chlorhexidine, I think for those patient who have been instructed on how to use it well, I've seen some positive outcome. But I've also noted several cases of skin-related reactions with chlorhexidine. So personally, I prefer the use of surgical spirit as opposed to chlorhexidine because for surgical spirit, you have not seen such reactions. But for chlorhexidine, we have noted such. Maybe something should be done to educate on how to apply so that the uptake can be improved.

3. Kindly describe the factors facilitating provision of chlorhexidine gel/solution

So I think the uptake of chlorhexidine would be practical when one, there is some medication around it, [inaudible] around it, bearing the pros and the cons of the use of the chlorhexidine. Two, this need to be integrated in the hospital procurement and also the hospital committee on procurement so that it can be availed in the pharmacist. And if it's available and we have done, or if we have used in a patient, then that would be easy to even convince the mothers to even purchase when they are getting discharged. So I think what is needed is a lot of education on it, practical use of it, and also assessment, and that will help in the uptake.

4. What Challenges do you experience with provision of chlorhexidine gel?

I think the greatest challenge on the access of chlorhexidine, one is I think the practice that has been there in the hospital because none of the practitioners actually recommend the use of this. And two, also availability, like in our pharmacy, either due to financial shortages or due to none-ordering of such medication [inaudible] chlorhexidine. It's not available in our facility. Neither do we get it even the pharmacy stocks around because in this facility, most of the drugs are not available, and patient are advised to buy around. But when you recommend such, they may not even getting around because it has not been on practice practically used.

5. What would you suggest would lead to an increase in use of CHX?

I think what will really increase the use of chlorhexidine in our facilities as earlier highlighted is public or desensitization of the health workers on the importance of use of chlorhexidine, the

side effect related with it and also the advantages that comes along with it. Two, it's the availability of the product in our facilities. If the hospital manager and the procurement committee put this as part of the essential drugs that need to be procured and it's available, I think the consumption and also the use by the patient will also improve. I think the third thing, I think we need generally a lot of sensitization around either in social media or also in terms of CME n the use of this so that it can be accepted our daily practice.

6. Comment on the IEC materials on CHX

In our facility, we have not had any information material on the application of chlorhexidine because it has not been largely used. However, that has been existing in the use of other... like TEO has been there. Also, the use of surgical spirit has been there. So I think this is a gap that we need to seal if this practice of use of chlorhexidine will have to be [inaudible] will have to be employed.

INDEX 23 – NAZARETH HOSPITAL

QUALITATIVE STUDY

A SEMI-STRUCTURED QUESTIONNAIRE

This questionnaire will be used to guide the interviewer.

Please let us record the following information that will be held confidentially and used only for the purpose of this research.

Bio data of the interviewee

Age…………………………… Professional line…………………….

Years of practice ……………..

Period of stay (years) in the specific station ……………………………

1. Please describe the existing cord care practices in our Kiambu County

From the day they severe the cord, within 24 hours, they use surgical spirit to clean the cord.

2. How would you describe your experience with CHX cord care antiseptic use in your hospital?

Most patients raise concern and healthcare workers raise concern of burning on the tissue. So mostly, they don't use it. They use surgical spirit.

3. Kindly describe the factors facilitating provision of chlorhexidine gel/solution

Availability of chlorhexidine, ease of application, and feedback from the patients.

4. What Challenges do you experience with provision of chlorhexidine gel?

Lack of chlorhexidine supply from KEMSA and also some of the staff are not comfortable using it due to patient concerns of soft tissue burning and also lack of knowledge on how to use it well.

5. What would you suggest would lead to an increase in use of CHX?

Train more healthcare workers on use, sensitize the public on the importance of chlorhexidine over the traditional surgical spirit, and also make it more available, especially in the centers.

6. Comment on the IEC materials on CHX

I have not seen it. It's not available.

INDEX 23 – NAZARETH HOSPITAL

QUALITATIVE STUDY

A SEMI-STRUCTURED QUESTIONNAIRE

This questionnaire will be used to guide the interviewer.

Please let us record the following information that will be held confidentially and used only for the purpose of this research.

Bio data of the interviewee

Age…………………………… Professional line…………………….

Years of practice ……………..

Period of stay (years) in the specific station ……………………………

1. Please describe the existing cord care practices in our Kiambu County

[inaudible] I think the first thing to use in cord care is surgical spirit.

Interviewer: So what about it?

Interviewee: It is effective according to me. Practically, it is effective because I'm a mother.

Interviewer: Okay.

2. How would you describe your experience with CHX cord care antiseptic use in your hospital?

That was information from the nursing department that it was causing sepsis. That is it. Yeah. It was not the best thing to use. Yeah.

3. Kindly describe the factors facilitating provision of chlorhexidine gel/solution

I won't say the factors that contributed. Of course now, what the thing I know, it was in excess, in surplus. The supply was in surplus. But the factors really, I did not know the factors which were used to supply chlorhexidine. You can't tell because I got it here. Yeah.

Interviewer: Was it used?

Interviewee: Yeah, it was used. Yes.

Interviewer: Result?

Interviewee: Not really effective. It was not really impressive. Yes.

Interviewer: Okay.

4. What Challenges do you experience with provision of chlorhexidine gel?

I cannot say that we have a challenge because now there was a time that... We get to a time that they were not in use anymore because of the complaint from the patient. Yeah.

Interviewer: Okay. So the challenge is that the need is not there.

Interviewee: The need is not there.

5. What would you suggest would lead to an increase in use of CHX?

I don't think there is need for increase because of the effect. Yeah.

6. Comment on the IEC materials on CHX

In the pharmacy department, we don't have any material as far chlorhexidine is concerned. Yes.

Interviewer: So would you like some?

Interviewee: *Yeah, we would like some to see what it contains.*

Interviewer: *Okay.*

Interviewee: *Yeah.*

[redacted]

INDEX 23 – NAZARETH HOSPITAL

QUALITATIVE STUDY

A SEMI-STRUCTURED QUESTIONNAIRE

This questionnaire will be used to guide the interviewer.

Please let us record the following information that will be held confidentially and used only for the purpose of this research.

Bio data of the interviewee

Age…………………………… Professional line…………………….

Years of practice ……………..

Period of stay (years) in the specific station ……………………………

1. **Please describe the existing cord care practices in our Kiambu County**

So once the cord has been cut and the umbilical clamp has been placed, the next thing is to maintain hygiene of the cord, and this is done by applying an antiseptic. Commonly we use chlorhexidine, either the gel or the solution. But other centers may use surgical spirit. Other centers may also use air to let it air dry naturally. So those are the methods that I'm aware that take care of the cord of the baby.

2. **How would you describe your experience with CHX cord care antiseptic use in your hospital?**

So chlorhexidine cord care has been beneficial in my experience to maintaining cord cleanliness after birth. Initially, we used to use the chlorhexidine gel, which was a bit difficult to use because it required one to wash their hands or change their gloves, apply the gel on their finger, and

then spread the gel on the cord and surrounding skin. The gel could also be confused with tetracycline eye ointment because of the kind of packaging it came in. However, with the solution, it's much easier to use because all one needs to do is to drop a few drops on the umbilical cord and leave the baby's cord to dry. The packaging is also very different from the tetracycline eye ointment, so confusion is reduced. We have also not seen an upsurge in cases of umbilical sepsis since we started using chlorhexidine solution regularly.

3. Kindly describe the factors facilitating provision of chlorhexidine gel/solution

So factors facilitating provision of chlorhexidine include demand. So there has to be a demand that has been created, especially by the healthcare workers because they're the ones who are going to use these antiseptics on the umbilical cords. So once a demand has been created, then the same is raised with the pharmacist who will then order and procure from the suppliers of medicines.

4. What Challenges do you experience with provision of chlorhexidine gel?

So challenges with provision of chloride gel include irregular supply. We've high months where the chlorhexidine is available. And then we have months where the chlorhexidine is unavailable mostly from the suppliers. Another challenge is that we may find some... The patient may not be able to purchase chlorhexidine to continue using at home.

5. What would you suggest would lead to an increase in use of CHX?

Healthcare workers, particularly midwives and doctors and pediatricians to be trained on the use of chlorhexidine and the benefits of chlorhexidine and studies to be conducted to prove the effectiveness of chlorhexidine. That would improve the uptake of chlorhexidine.

6. Comment on the IEC materials on CHX

So I've seen IEC materials only in the media, online, but I've not seen, a physical chart provided for the hospital to use chlorhexidine. So physical charts will be beneficial.

INDEX 23 – NAZARETH HOSPITAL

QUALITATIVE STUDY

A SEMI-STRUCTURED QUESTIONNAIRE

This questionnaire will be used to guide the interviewer.

Please let us record the following information that will be held confidentially and used only for the purpose of this research.

Bio data of the interviewee

Age…………………………… Professional line…………………….

Years of practice ……………..

Period of stay (years) in the specific station ……………………………

1. Please describe the existing cord care practices in our Kiambu County

So currently in Kiambu County, what we are advocating for cord care is the use of the chlorhexidine, and specifically the chlorhexidine gel. We advocate that we use the 7.1%, which delivers 4% chlorhexidine to the cord. Chlorhexidine cord care should actually be started at birth. Once the baby is born and the cord is cut, the chlorhexidine should be applied on the cord stamp. It should also be applied around the cord. Subsequently the mother should be taught how to use the chlorhexidine once a day for at least seven days.

2. How would you describe your experience with CHX cord care antiseptic use in your hospital?

So currently in Kiambu County, what we are advocating for cord care is the use of the chlorhexidine, and specifically the chlorhexidine gel. We advocate that we use the 7.1%, which

delivers 4% chlorhexidine to the cord. Chlorhexidine cord care should actually be started at birth. Once the baby is born and the cord is cut, the chlorhexidine should be applied on the cord stamp. It should also be applied around the cord. Subsequently the mother should be taught how to use the chlorhexidine once a day for at least seven days.

3. Kindly describe the factors facilitating provision of chlorhexidine gel/solution

So currently in Kiambu County, what we are advocating for cord care is the use of the chlorhexidine, and specifically the chlorhexidine gel. We advocate that we use the 7.1%, which delivers 4% chlorhexidine to the cord. Chlorhexidine cord care should actually be started at birth. Once the baby is born and the cord is cut, the chlorhexidine should be applied on the cord stamp. It should also be applied around the cord. Subsequently the mother should be taught how to use the chlorhexidine once a day for at least seven days.

4. What Challenges do you experience with provision of chlorhexidine gel?

So currently in Kiambu County, what we are advocating for cord care is the use of the chlorhexidine, and specifically the chlorhexidine gel. We advocate that we use the 7.1%, which delivers 4% chlorhexidine to the cord. Chlorhexidine cord care should actually be started at birth. Once the baby is born and the cord is cut, the chlorhexidine should be applied on the cord stamp. It should also be applied around the cord. Subsequently the mother should be taught how to use the chlorhexidine once a day for at least seven days.

5. What would you suggest would lead to an increase in use of CHX?

I think some of the things that would cause an increase in use of chlorhexidine is first increasing the scope of training to the healthcare workers so that they are knowledgeable. Still we have a huge gap. Many healthcare workers do not know about chlorhexidine gel for cord. So we need to increase the scope of our training. Also, as healthcare workers, we need to be more acceptable and adaptable to new forms of treatment and technology, which are out there, not necessarily sticking to what we know about the past, like use of the methylated spirit. Also, we need to increase availability of chlorhexidine gel at all levels of healthcare facilities so that even though a mother delivers at home and presents to a dispensary or health center, after two to

three days, the chlorhexidine should be available for her to use. We can also use the patient education and media outlets in order to educate our mothers on the importance of chlorhexidine gel in prevention of cord sepsis.

6. Comment on the IEC materials on CHX

Okay. We do have some IEC materials which are available. We have some posters which we have available in our maternity wards, in our newborn unit, especially the labor ward because we emphasize that the chlorhexidine should be applied immediately the baby is born. So we do have some posters which are available, and they usually show the step-by-step technique on how to apply the chlorhexidine on the cord. That's the only material that we have available.

Interviewer: Do you have enough?

Interviewee: I would not say that there's enough. I think we need more.

INDEX 23 – NAZARETH HOSPITAL

QUALITATIVE STUDY

A SEMI-STRUCTURED QUESTIONNAIRE

This questionnaire will be used to guide the interviewer.

Please let us record the following information that will be held confidentially and used only for the purpose of this research.

Bio data of the interviewee

Age………………………… **Professional line…………………….**

Years of practice ……………..

Period of stay (years) in the specific station ……………………………

1. Please describe the existing cord care practices in our Kiambu County

I would describe the usage of Cchlorhexidine immediately after the birth of foetus, we give chlorhexidine when it is available. At times it is not available in the facility.

Interviewer: But how do you give it?

Interviewee: In the case of dosage, we just apply once? Yes.

2. How would you describe your experience with CHX cord care antiseptic use in your hospital?

In the hospital, I would describe the use of chlorhexidine as effective immediately when the mother has given birth. Yes. It is effective, immediately.

3. Kindly describe the factors facilitating provision of chlorhexidine gel/solution

It is effective, first of all, and the second comment I can talk about chlorhexidine on this question is that the health workers are appreciating it. So they're using it in the hospital

Interviewer: And is available?

Interviewee: Yeah. When is available. And at times it is not. But when it is, we appreciate it. Yes.

Interviewer: So you can say all health workers have been taught about it?

Interviewee: Not really. Because sometimes you just give because it is there, and it is effective. Yes.

Interviewer: So when is not, what do you do?

Interviewee: We don't give. Yes. We ask the mothers to buy.

4. What Challenges do you experience with provision of chlorhexidine gel?

I'll talk about demand because most of the time you find that the drug is not readily available in the hospital. And if it is not ready in the hospital, definitely it won't be found in other pharmacies or chemists. Also, I'll talk about ignorance from the mothers. And another thing is also talk about tradition. Some community don't believe in medication or the use of chlorhexidine in the hospital. Yes.

Interviewer: But what of the healthcare workers?

Interviewee: The healthcare workers, they don't have any problem with it. Yes.

5. What would you suggest would lead to an increase in use of CHX?

I'll talk about availability. When chlorhexidine is available to the hospital, mothers should be able to access the drug. I'll also talk about health education to their mothers. The mothers should be taught on the importance of the drugs. Also, to our healthcare workers. They're also

supposed to be educated about the drugs, the contraindication, the indications, all of them, so that they can be able to explain to the mothers when they're administering. And also, I'll talk about the price. For instance, the drug is not available in our facility, at least they can be able to access it in other chemist and pharmacists.

6. Comment on the IEC materials on CHX

I'll talk of its availability and adequacy of the IEC materials so that the health workers are able to see them and read about and be encouraged about them. Yes.

Interviewer: So are you having the materials?

Interviewee: Not enough.

Interviewer: No.

Interviewee: Talk of it. Yes.

INDEX 23 – NAZARETH HOSPITAL

QUALITATIVE STUDY

A SEMI-STRUCTURED QUESTIONNAIRE

This questionnaire will be used to guide the interviewer.

Please let us record the following information that will be held confidentially and used only for the purpose of this research.

Bio data of the interviewee

Age…………………………… Professional line…………………….

Years of practice ……………..

Period of stay (years) in the specific station ……………………………..

1. Please describe the existing cord care practices in our Kiambu County

So after delivery, we use the chlorhexidine for cord care. We usually give them the baby stat dose. But when they're available in large quantities, we usually give the mother to stay with the drug itself so they can apply it twice per day.

Interviewer: Okay. Twice per day?

Interviewee: Yeah. Twice.

2. How would you describe your experience with CHX cord care antiseptic use in your hospital?

So far, the outcomes have been good. We have had limited cases of sepsis after the use of chlorhexidine.

3. Kindly describe the factors facilitating provision of chlorhexidine gel/solution

So the chlorhexidine is usually sourced by the hospital. So sometimes the challenge that we have, if they had sourced and it is limited in quantity, we usually give as a stat dose. If there's enough, then we give every mother individually, they have one until discharge.

4. What Challenges do you experience with provision of chlorhexidine gel?

Sometimes there's shortage. So we have to inform them. We have to inform the mothers to source the chlorhexidine from outside. And sometimes considering that some of the patients are limited in resources, some of them cannot access the drug.

5. What would you suggest would lead to an increase in use of CHX?

Suggest that the hospital should source for more of the drug to ensure that it's constantly available for use to increase its uptake.

Interviewer: If it was available, do you think everybody would use.

Interviewee: Yeah.

Interviewer: Okay.

Interviewee: Availability is the issue.

6. Comment on the IEC materials on CHX

The amount of information charts we have are limited. We could benefit from an increase in the charts, the IEC materials.

INDEX 23 – NAZARETH HOSPITAL

QUALITATIVE STUDY

A SEMI-STRUCTURED QUESTIONNAIRE

This questionnaire will be used to guide the interviewer.

Please let us record the following information that will be held confidentially and used only for the purpose of this research.

Bio data of the interviewee

Age………………………… **Professional line…………………….**

Years of practice ……………..

Period of stay (years) in the specific station ……………………………

1. Please describe the existing cord care practices in our Kiambu County

In Kiambu, we normally use chlorhexidine gel for cord care. It's given at birth, and sometimes the mothers are given the chlorhexidine gel to use at home. There are times when the chlorhexidine gel is out of stock. Then the patients are normally advised to use the spirit, which doesn't work as well.

2. How would you describe your experience with CHX cord care antiseptic use in your hospital?

The use of chlorhexidine for cord care has made work easier in Kiambu since it's easy to use and its availability, its efficacy being high has made it easier for us to manage the newborns and their mothers are even happy with the healing process using chlorhexidine gel.

3. Kindly describe the factors facilitating provision of chlorhexidine gel/solution

The first reason why we have chlorhexidine gel is because of demand. And we normally get it through the access program normally. Or sometimes when the access program cannot be able to give us that, then the hospital has to give money at least on a quarterly basis so that we can buy chlorhexidine gel. I can't say that it's provided all the time because there are times that we lack. But normally, the demand is what makes us to run up and down to get the chlorhexidine gel.

4. What Challenges do you experience with provision of chlorhexidine gel?

The first problem we normally experience in providing chlorhexidine gel is that our funds sometimes delay as we get funds from the county for buying the chlorhexidine gel. So when the funds delay, then we remain out of stock of chlorhexidine gel for quite a long time. And there is a time that the molecule chlorhexidine for chlorhexidine gel was not available, sometime in the beginning of the year. We also suffered. We couldn't source from anywhere. So basically those are the two challenges we have, when it's out of stock, and sometimes when you don't have funds to buy.

5. What would you suggest would lead to an increase in use of CHX?

What can lead into an increased use of chlorhexidine gel now that we can see it's working is that the mothers need an education. There are mothers who will not want to use chlorhexidine gel because of mythical thoughts or superstition, or that's not what they were taught by their mothers to use. So sensitization is needed. And also, sensitization among the users, those are the mothers, is needed. And also, we need to sensitize the healthcare workers because sometimes we have found mothers who have not used chlorhexidine gel because the physician who was working with the mother did not believe in the working of chlorhexidine gel. So I think even the healthcare workers need also to be told or to be taught, to be sensitized about chlorhexidine gel.

6. Comment on the IEC materials on CHX

In my opinion, the IEC material available for chlorhexidine gel do not display what its work is. They don't encourage the use of it as much. I feel like we could do more to encourage, to put strong words towards its use because from the material, the readers, both the users, the mothers, and prescribers might not see how efficacious chlorhexidine gel is. And the material should be available in our outpatient clinics, MCH. And also, I think we need to have healthcare workers also handed over this material personally and sometimes maybe stuck on the wards, like now the newborn unit, where it's normally used. As a person passes by, then reads about it, they can be reminded about it. So the supply... No, the availability of this material to the right people I think is what needs to be done, in my opinion.

INDEX 23 – NAZARETH HOSPITAL

QUALITATIVE STUDY

A SEMI-STRUCTURED QUESTIONNAIRE

This questionnaire will be used to guide the interviewer.

Please let us record the following information that will be held confidentially and used only for the purpose of this research.

Bio data of the interviewee

Age…………………………… Professional line…………………….

Years of practice ……………..

Period of stay (years) in the specific station ……………………………

1. Please describe the existing cord care practices in our Kiambu County

So immediately we cut the cord, that is when we detach the mother and the baby, we clump it using a cord clump. Then after that, we do the first application of the chlorhexidine gel on the baby. Then from there, the mother now goes with the baby to postnatal. From there, there is health talk on how to take care of the cord. The mother is given. We are told to... We tell them to buy the chlorhexidine gel itself. That's what we use here. They apply it once a day for seven days. That is what we do.

2. How would you describe your experience with CHX cord care antiseptic use in your hospital?

Well, I would say the experience with chlorhexidine gel is quite okay, the gel itself. We have had some issues with the drops specifically. But with the gel, we are okay with it. The results are

okay. But we have to get to look at the drops. I don't know where the difference is coming in. But with the gel, we are getting really nice results.

Interviewer: What challenges do you have with the drops?

Interviewee: The drops, the mothers are coming back with septic cords. I don't know whether it's the hygiene at home or what. I don't get. But when we get a mother that comes and then we take the history, they say they bought the drops. But with the gel, no, not much sepsis. But with the drops, there is.

3. Kindly describe the factors facilitating provision of chlorhexidine gel/solution

The first factor is the uptake is positive, that makes it be used, that's the gel. Two, it's the availability. It's really not available in our facility, but it is readily available outside. Those are the factors that we have.

4. What Challenges do you experience with provision of chlorhexidine gel?

The first challenge that we have with the gel is that it's not available in our facility. The mothers have to buy, so it's an extra cost for the mother. That is the biggest challenge that we have. If we get it available in our facility, then we'll be good.

5. What would you suggest would lead to an increase in use of CHX?

The first one is make it available. Let it be charged as a package, the way the mother comes and then they're told they have a whole package, let it be factored in to be used whether in Linda Mama or NHIF. That will be easier for it. Then the hospital to make it readily available. It is easier to access it in the hospital than outside. So if it is readily available, that would be really nice and covered by the packages that we have here, the insurance that we have here at the hospital. If it is covered total and the mother comes in and when the mother gives birth, they're just given and go with it at home, that would really help.

6. Comment on the IEC materials on CHX

Well, we would ask for an increase, our provision of IEC materials in the wards. There are very few. And some places, we do not have on specifically cord care. So the IEC materials should be provided.

Interviewer: So at the moment, did you have any?

Interviewee: No. At our ward, we do not have.

Interviewer: But you have seen one?

Interviewee: Yes, I've seen one. I've seen one.

Interviewer: Elsewhere.

Interviewee: Yes. Elsewhere. In the pediatric ward, I think there is. And then in the newborn unit, there is. We did some in the postnatal as well.

Interviewer: What would you commend about the quality?

Interviewee: The quality of?

Interviewer: The IEC materials.

Interviewee: Let it be clear and understandable to the normal mwananchi. Let it be clear to the mothers in a language they can understand and read. Yes.

INDEX 23 – NAZARETH HOSPITAL

QUALITATIVE STUDY

A SEMI-STRUCTURED QUESTIONNAIRE

This questionnaire will be used to guide the interviewer.

Please let us record the following information that will be held confidentially and used only for the purpose of this research.

Bio data of the interviewee

Age…………………………… Professional line…………………….

Years of practice ……………..

Period of stay (years) in the specific station ……………………………..

1. Please describe the existing cord care practices in our Kiambu County

You just dispense to them, to the maternity. You just give to the maternity, and they tell the patient how to use. So they're the one who interact with the patient directly. Yes.

Interviewer: So you don't know the cord care practice in Kiambu County?

Interviewee: I just know most of the time, they don't even use that chlorhexidine. They use surgical spirit. Yes.

2. How would you describe your experience with CHX cord care antiseptic use in your hospital?

I have never used it.

3. Kindly describe the factors facilitating provision of chlorhexidine gel/solution

Mostly in Kihara, they don't use that a lot. They just use surgical spirit. It's rare for me to dispense that.

4. What Challenges do you experience with provision of chlorhexidine gel?

So we rarely miss them. We rarely demand for them. They're always there. We don't have a shortage.

5. What would you suggest would lead to an increase in use of CHX?

It's not as effective compared to surgical spirit. So if they're able to make it more effective, as in, it can show results way better than surgical spirit, then it means the demand will be higher. Because there must be a reason why it is not always recommended by nurses because they're the one who interact with the patients, they're the ones who interact with the kids. So if they have been using it for some time and it is not working, there is reason why we usually switch to surgical spirit. So I can't answer that directly, but what I know is there must be something that is making them not use it. Maybe it is not as effective in healing the wound of the baby.

6. Comment on the IEC materials on CHX

I have not seen any charts describing the use of chlorhexidine gel. But maybe if they're provided, then we need to have some more information on it and how it is used.

INDEX 23 – NAZARETH HOSPITAL

QUALITATIVE STUDY

A SEMI-STRUCTURED QUESTIONNAIRE

This questionnaire will be used to guide the interviewer.

Please let us record the following information that will be held confidentially and used only for the purpose of this research.

Bio data of the interviewee

Age………………………… **Professional line…………………….**

Years of practice ……………..

Period of stay (years) in the specific station ……………………………

1. Please describe the existing cord care practices in our Kiambu County

So my understanding of cord in Kiambu County, patients are advised about hygiene, that is, cleaning of the cord. In some places, they use surgical spirit for cleaning, for instance, in Kihara Level 4. I've also seen some places use chlorhexidine, both the gel and the solution. But most nurses prefer using the solution. I'm yet to know why.

2. How would you describe your experience with CHX cord care antiseptic use in your hospital?

So my experience with the antiseptics, I think the uptake is generally quite good. I have not experienced any patients come back with infections. And also, on the healthcare workers, I think they have a positive attitude towards use of antiseptics in the management of cord infections.

Interviewer: *Are you specific on chlorhexidine or?*

Interviewee: *I'm not specific on chlorhexidine. Because again, we have challenges on availability of chlorhexidine. It's not every time that we have chlorhexidine at the pharmacy. So I'm being general on all antiseptics.*

3. Kindly describe the factors facilitating provision of chlorhexidine gel/solution

Okay. So the factors facilitating provision of chlorhexidine. Of course, the demand is there from the patients. Since price is based on the outcome, that is the report we receive from the patients who've used chlorhexidine before. They have positive reports, in that, they don't get infections. Their babies don't get infections, cord infections. Again, about facilitation, on availability. I don't think the facilitation is that good. At some point, we run out of the drugs, that is, chlorhexidine, from the healthcare workers. Also, they have a positive report concerning chlorhexidine, in that, after prescribing the drug, they're able to receive a positive outcome from their patients.

4. What Challenges do you experience with provision of chlorhexidine gel?

The first challenge is limited resources from the county. That is, when receiving our delivery from KEMSA, we might also be able to receive chlorhexidine. Another challenge is from the patients. Once the drug is prescribed, some patients may not be able to purchase from the pharmacies outside. Another challenge may be the population when the demand for the solution is more compared to the gel. So we would prefer to have the solution. I think that's all.

5. What would you suggest would lead to an increase in use of CHX?

First is training healthcare workers of the importance of use of chlorhexidine gel for the patients. Also, educating the patients on the use of chlorhexidine gel and the importance of it in infection prevention. Also, ensuring the gel is available in our facilities would lead to increased use of the drug.

6. Comment on the IEC materials on CHX

Concerning the IEC materials in our station have not interacted with them. But if they were there, they would be a constant reminder both to the healthcare worker and to the patient on the use of chlorhexidine. So I wouldn't have much to comment about it since they're not available in our facilities. Thank you.

INDEX 23 – NAZARETH HOSPITAL

QUALITATIVE STUDY

A SEMI-STRUCTURED QUESTIONNAIRE

This questionnaire will be used to guide the interviewer.

Please let us record the following information that will be held confidentially and used only for the purpose of this research.

Bio data of the interviewee

Age………………………… **Professional line…………………….**

Years of practice ……………..

Period of stay (years) in the specific station ……………………………

1. Please describe the existing cord care practices in our Kiambu County

So for our mothers, when they come because us, we interact them from two weeks, most of the mothers that come, they're usually on chlorhexidine, although some come that are using surgical spirit. So I would say in Kiambu County, we are usig both the chlorhexidine gel and the surgical spirit.

2. How would you describe your experience with CHX cord care antiseptic use in your hospital?

So for me, the use of chlorhexidine, the mothers that I've seen, most of them, at two weeks, the cord usually has not fallen off. It's still wet. And most of the time when the cord has not fallen at two weeks, we usually sent them to be seen by the clinician, and they're usually put on surgical spirit. So my experience with chlorhexidine, I would say the cord takes time to heal.

Interviewer: *Do you use chlorhexidine gel or solution?*

Interviewee: *Gel.*

3. Kindly describe the factors facilitating provision of chlorhexidine gel/solution

I know the chlorhexidine is usually given at maternity when mothers deliver or when they're being discharged. I know it is in pharmacy stores and also in our pharmacy, but I don't know whether it's given for free or the mothers buy.

4. What Challenges do you experience with provision of chlorhexidine gel?

I think the fact that most of the healthcare workers were used to surgical spirit, the switch to chlorhexidine and the fact that it takes time, the cord takes time to heal, I think most people are still reluctant to start mothers on the gel. They'll prefer surgical spirit.

Interviewer: *Have you had any training on chlorhexidine?*

Interviewee: *Yes, I've had a training. Usually, when we take our monthly reports, we usually get training. Sometimes you can get training on the use of chlorhexidine, sometimes on [inaudible].*

5. What would you suggest would lead to an increase in use of CHX?

I think we need proper training for the staff, especially in maternity, even for us working in the postnatal clinics, because I understand the gel should be started immediately after birth. So maybe that might be one of the contributing factors as to why the cord does not... It takes time to heal.

6. Comment on the IEC materials on CHX

I have not seen any. But the fact that I work at the postnatal clinic and have not seen any material.

Interviewer: *So the materials are not available in Kihara?*

Interviewee: *They're not available.*

INDEX 23 – NAZARETH HOSPITAL

QUALITATIVE STUDY

A SEMI-STRUCTURED QUESTIONNAIRE

This questionnaire will be used to guide the interviewer.

Please let us record the following information that will be held confidentially and used only for the purpose of this research.

Bio data of the interviewee

Age…………………………… Professional line…………………….

Years of practice ……………..

Period of stay (years) in the specific station ……………………………..

1. Please describe the existing cord care practices in our Kiambu County

Okay. We do cord care by using chlorhexidine. Sometimes we use the surgical spirit when chlorhexidine is not present.

Interviewer: Anything else?

Interviewee: Nothing.

Interviewer: Okay. What do you think is recommended by Kiambu County?

Interviewee: Chlorhexidine.

2. How would you describe your experience with CHX cord care antiseptic use in your hospital?

It's been picked well, and it's working well. We have at least few mothers who come with sepsis afterwards, but when you ask them, maybe the hygiene was not good. But those who practice, okay, cord care with chlorhexidine as they are instructed, the outcome is good.

3. Kindly describe the factors facilitating provision of chlorhexidine gel/solution

One I think is the resources and the support from the county. Yeah. So you know we get our nini from KEMSA, which is being facilitated from the [inaudible]. So if we don't have, it becomes a problem because we do teach mothers on how to use, but sometimes they don't go and buy, which is not good.

Interviewer: Do they buy when you tell them to buy?

Interviewee: They buy because we make it as a requirement for every mother to go home with it.

4. What Challenges do you experience with provision of chlorhexidine gel?

And now mothers understanding.

Interviewer: Start again. Start again.

Interviewee: Okay. Availability of chlorhexidine, and the challenges again is some mothers do not take it, as in, you can explain to the mother, okay, the benefits, but after going home, she does his things. think there are some things they do like spiting on the cord and such like things.

5. What would you suggest would lead to an increase in use of CHX?

The availability of chlorhexidine and the awareness to healthcare workers and mothers.

Interviewer: What do you mean by awareness?

Interviewee: Mothers should be taught. You know they were used with traditional things and surgical spirit. So their awareness of chlorhexidine. You know there are mothers who don't know aboutit.

Interviewer: What of you as health workers, have you been trained?

Interviewee: *No, it was just introducing, but others were trained.*

6. Comment on the IEC materials on CHX

Okay. With chlorhexidine or which materials? chlorhexidine, no. They are not.

Interviewer: *You don't have any in the facility?*

Interviewee: *No. No.*

Interviewer: *Have you ever seen any?*

Interviewee: *No.*

INDEX 23 – NAZARETH HOSPITAL

QUALITATIVE STUDY

A SEMI-STRUCTURED QUESTIONNAIRE

This questionnaire will be used to guide the interviewer.

Please let us record the following information that will be held confidentially and used only for the purpose of this research.

Bio data of the interviewee

Age…………………………… Professional line…………………….

Years of practice ………………

Period of stay (years) in the specific station ……………………………

1. Please describe the existing cord care practices in our Kiambu County

The cord care we give, when the mother delivers, for the updates, we have the chlorhexidne. They're given and they apply immediately. But we also have the surgical spirit, which they apply for the cord care.

Interviewer: So do they apply both?

Interviewee: No, they don't apply both. The ones who are given chlorhexidine if it's available, they apply. But the ones who don't have, they're given surgical spirit for cord care.

2. How would you describe your experience with CHX cord care antiseptic use in your hospital?

I've said for postnatal, the ones who applied immediately after birth, it is working well. But for the ones who didn't use it immediately, the cord still it-- They say-

Interviewer: Just say it.

Interviewee: It takes a longer time before-- you say before it falls ama before it heals if the mother does not apply chlorhexidine immediately in the maternity?

Interviewer: What do you mean by immediately?

Interviewee: Immediately after birth.

Interviewer: Okay. Is it a few minutes, a few hours?

Interviewee: I'm not sure.

3. Kindly describe the factors facilitating provision of chlorhexidine gel/solution

Factors that influence the provision of the chlorhexidine, one of them, like now, I'm working in the antenatal clinic. For us here, we don't provide. But for the ones who are in maternity, they provide the chlorhexidine. But for us in the ANC, we don't give the mothers the chlorhexidine.

4. What Challenges do you experience with provision of chlorhexidine gel?

Okay. In the antenatal clinic, here, we are not provided with the chlorhexidine. But if we are provided with, we're going to give to our mothers.

5. What would you suggest would lead to an increase in use of CHX?

The first one is the provision of the chlorhexidine. If we are provided with them, they come, they issue the mothers and also the health education. Give more knowledge, educate more about the chlorhexidine.

6. Comment on the IEC materials on CHX

And materials, actually, I myself, I've never seen any materials for chlorhexidine in our team, that is ANC.

INDEX 23 – NAZARETH HOSPITAL

QUALITATIVE STUDY

A SEMI-STRUCTURED QUESTIONNAIRE

This questionnaire will be used to guide the interviewer.

Please let us record the following information that will be held confidentially and used only for the purpose of this research.

Bio data of the interviewee

Age………………………… **Professional line…………………….**

Years of practice ……………..

Period of stay (years) in the specific station ……………………………

1. Please describe the existing cord care practices in our Kiambu County

So after birth, when you cut the cord, immediately we use the chlorhexidine, and after, we use it once a day, once a day after delivery until now the mothers goes home and you instruct them to use it until the cord dries off and falls off.

2. How would you describe your experience with CHX cord care antiseptic use in your hospital?

My experience with chlorhexidine is that it is much better than the spirit used to use before. I think it's much better, for me. The cords are drying up faster than the spirit.

3. Kindly describe the factors facilitating provision of chlorhexidine gel/solution

Yes. So number one, it's the cost. It is readily available to them considering that it is covered by NHIF, number one. So the matter of the cost is out of the question. Number two, it's the use of application. So when you compare with the spirit, the chlorhexidine will just squeeze out of the tube compared to the what, to the spirit and the cotton, hiyo process yote. Then another factor is the patients are enlightened about the use of chlorhexidine. So before even you educate them on the use of it, they already know that there's a katube that they should be given for the cord care. So another factor is I think technology. So for example, primigravida are usually anxious. So they usually know. Wanakuanga wameulizia ulizia, then wamegoogle google. So I think they usually know before hata wapewe. So they usually look forward to be given the chlorhexidine. It's rare upate mtu akikwambia story na, inaitwa aje, spirit.

4. What Challenges do you experience with provision of chlorhexidine gel?

So with the challenges that we are getting is that the tube is small. So you find that it is getting finished before the cords falls off. The second point is that some mothers are resistant to change, especially those who delivered much earlier and chlorhexidine was not there before. So if they used spirit, they still want to use spirit or maybe some other techniques that they used to use. I think that's all.

Interviewer: What are these other techniques.

Interviewee: Like using saliva or cow dung. Yeah. Yeah. There are those in the community that use saliva or cow dung.

5. What would you suggest would lead to an increase in use of CHX?

So creating awareness for the mothers, giving them health messages to know about chlorhexidine and also the health workers also to be educated on the chlorhexidine, and maybe reduce the cost because we say the tube is very small, and maybe there are some mothers who can use to a maximum of two. They can reduce the cost for the tube.

6. Comment on the IEC materials on CHX

For the charts, they should be bigger, and they should provide more charts so that we can put maybe in the wards and to educate the mothers. And for those mothers who are illiterate who cannot read, the wordings are many than the pictures. The pictures are better illustrating of how to use the chlorhexidine. That would be best to include all the mothers.

INDEX 23 – NAZARETH HOSPITAL

QUALITATIVE STUDY

A SEMI-STRUCTURED QUESTIONNAIRE

This questionnaire will be used to guide the interviewer.

Please let us record the following information that will be held confidentially and used only for the purpose of this research.

Bio data of the interviewee

Age………………………… **Professional line…………………….**

Years of practice ……………..

Period of stay (years) in the specific station ……………………………

1. Please describe the existing cord care practices in our Kiambu County

So the existing cord care practice, specifically in our facility is the use of the chlorhexidine cream, which is applied immediately after birth, after the cord is cut, then the chlorhexidine is applied. Then thereafter, it is applied once a day till the cord falls off. But in case the cord falls off before the chlorhexidine is over, the mothers usually stop the use of the chlorhexidine cream.

2. How would you describe your experience with CHX cord care antiseptic use in your hospital?

So far, we've had a good experience with the use of chlorhexidine cream because we rarely get a baby come back with a cord care infection ama septic cord, all things related to it. Yes, yes, yes.

3. Kindly describe the factors facilitating provision of chlorhexidine gel/solution

Factor number one, it's the policy of the hospital to purchase the chlorhexidine cream for the cord care. Number two, it is the cost. So a tube goes for around 120 shillings, which is averagely affordable for most Kenyans. Number two, it's the factor of the NHIF cover. It counts with the cover, so it is free to them. Number three, it's the factor of awareness to the client.

4. What Challenges do you experience with provision of chlorhexidine gel?

So challenge number one is the tube is small. So most of the time, the chlorhexidine is finished before the cord is cut off, ama it drops. Haya. Number two, nimeformat.

5. What would you suggest would lead to an increase in use of CHX?

Number one, doing a community outreach to create awareness to those population who do not know about the chlorhexidine cream. Number two is to try and explain to the clients about the advantages of using that specific commodity. Then number three, it's to-

6. Comment on the IEC materials on CHX

Number one, to increase its provision because in our facility, we only have one chat. Number two, they should do more of picture demonstration than the wordings because of the illiteracy of some clients who cannot read and understand what it says.

INDEX 23 – NAZARETH HOSPITAL

QUALITATIVE STUDY

A SEMI-STRUCTURED QUESTIONNAIRE

This questionnaire will be used to guide the interviewer.

Please let us record the following information that will be held confidentially and used only for the purpose of this research.

Bio data of the interviewee

Age…………………………   **Professional line…………………….**

Years of practice ……………..

Period of stay (years) in the specific station ……………………………

1. Please describe the existing cord care practices in our Kiambu County

Well, in Kiambu County, we use chlorhexidine as the mode of cord care after delivery of mothers of the babies.

Interviewer: At what time do you give it?

Interviewee: We give it immediately after birth. Then we show the mothers how to use it when they go home and how to use it even at home.

2. How would you describe your experience with CHX cord care antiseptic use in your hospital?

Okay. The uptake is good for the chlorhexidine because it enhances healing of the cord, and it has also reduced infection to the mothers. The mothers don't come back here with infected cords. Yeah. The uptake is good.

3. Kindly describe the factors facilitating provision of chlorhexidine gel/solution

Factors enhancing proper use of chlorhexidine. One, we start with here in Nazareth Hospital, all the mothers are ordered for, whether you are cash paying, whether you are check paying, you're ordered for upon delivery, and then it is charged later. The mother pays later for it. So we don't have issues with the provision. It is available in our pharmacy. The other thing, the mothers are knowledgeable, and they understand quickly how to use it, and they also do return demonstration on how to use it. The other factor... Probably we can say about the two as I think of others later.

4. What Challenges do you experience with provision of chlorhexidine gel?

One challenge is that mothers out there, they have other methods of doing cord care. When you teach them how to do it, they go at home and they're taught by other relatives how to do it. So you find them mixing with other things. Like they will buy spirit because the other baby they used spirit, or they will use another thing like savlon that's hindering proper use of the chlorhexidine.

5. What would you suggest would lead to an increase in use of CHX?

First, I would recommend it for the prices of the one tube of the chlorhexidine to be lowered so that it can be easily accessible to all the mothers. Secondly is proper training of the staff so that they can teach also properly to the mothers, and also follow-up for the mothers at home on how we can do it, whether they're doing it properly, and how they're going with it at home.

6. Comment on the IEC materials on CHX

On the materials for the chlorhexidine, I would recommend first they be available to the postnatal rooms and antenatal rooms so that as the mothers come, they are aware there are new methods of doing the cord care. Then the pictures and the lighting to be seen, to be of good quality so that when you are far, you can also read. Yeah. And the materials also to be everywhere, in every ward, in the postnatal rooms, and in labor ward, and also here in antenatal.

INDEX 23 – NAZARETH HOSPITAL

QUALITATIVE STUDY

A SEMI-STRUCTURED QUESTIONNAIRE

This questionnaire will be used to guide the interviewer.

Please let us record the following information that will be held confidentially and used only for the purpose of this research.

Bio data of the interviewee

Age…………………………… Professional line…………………….

Years of practice ……………..

Period of stay (years) in the specific station ……………………………

1. **Please describe the existing cord care practices in our Kiambu County**

Okay. The cord care in Kiambu County, it starts from when it is cut from the mother, the baby is separated from the mother, and it is clumped. Then from there, we apply either the chlorhexidine. Previously used to do with the methylated spirit. But since the year, it was from 2016, they introduced the use of the chlorhexidine gel. And so far, it's doing good, though there are some mothers who don't know how to do the care, and we advise them to do twice daily, and they make sure that they do it around the cord. Though there are some mothers who don't know how to do it. They just apply. They cover it immediately. Because sometimes they still get that risk of the infection. They come back after two weeks or so. They come back with sepsis. Yeah.

2. How would you describe your experience with CHX cord care antiseptic use in your hospital?

The use of chlorhexidine cord care, the 7.1, so far, it's good because the risk of sepsis, the mothers have not been complaining so much, except for the few. The results are good because it's within some days, the cord is cord is already cut off, maybe a week.

3. Kindly describe the factors facilitating provision of chlorhexidine gel/solution

So the factors that have affected the provision of the chlorhexidine in our hospital, first of all, we have the policy that is an order that came from Kiambu County from the Ministry of Health that chlorhexidine should be used. We should stop using the methylated spirit because it's the safest. And also, we have so many mothers that we buy more of them. And again, another one is...

Interviewer: Are they able to buy?

Interviewee: They're able to buy because most of it's under the NHIF. So we charge it under NHIF, and it's also cost-effective.

4. What Challenges do you experience with provision of chlorhexidine gel?

Okay. The challenges that we are having with the chlorhexidine, maybe they're out of stock when it comes out of stock from where we buy and maybe some mothers do not know how to use it. So they'll come back and say it's not working. So the prescriber will have to change... They change from gel to solution because I think maybe the gel is not being used well. So some of the prescribers, they interchange it with the solution and also [inaudible].

5. What would you suggest would lead to an increase in use of CHX?

What will increase the use of chlorhexidine, first of all, we educate mothers more, or we create awareness on the importance of chlorhexidine compared to other compared to other nini that are used, solutions that are used to do with cord care and mostly education and creating awareness, especially from the antenatal. The postnatal, they do most of educating mothers on how to use it. So if the mother know how to use it, I think they'll always spell the gospel that I used the chlorhexidine, and it really worked well.

6. Comment on the IEC materials on CHX

The availability of IECs in our hospital, because I've seen it from the MCH, the charts are clear, though... They're clear. And even if the mothers, they have a look at it, they will be able to understand. The availability of them, we don't have much, because it's only in the MCH where we can only see. And as from our department, we also need such charts so that we can also how to tell them. Because some come from the outpatient part. So we need also to know. But the availability of it within our hospital is not that much.

INDEX 23 – NAZARETH HOSPITAL

QUALITATIVE STUDY

A SEMI-STRUCTURED QUESTIONNAIRE

This questionnaire will be used to guide the interviewer.

Please let us record the following information that will be held confidentially and used only for the purpose of this research.

Bio data of the interviewee

Age…………………………… Professional line…………………….

Years of practice ……………..

Period of stay (years) in the specific station ……………………………

1. Please describe the existing cord care practices in our Kiambu County

The cord care practices that we use here, there's chlorhexidine digluconate, which the mothers apply, and the mothers apply. Then we review them. Immediately after birth, they start applying it. Then we review them in the clinics after two weeks after discharge, whereby we see whether the cord has fallen off or not. If the cord is clean. If there's a bit of... If the cord is not clean, we encourage them to change to Hexicod drops now.

2. How would you describe your experience with CHX cord care antiseptic use in your hospital?

About experience with chlorhexidine digluconate, I would say that, for some mothers, it works well, and the cord actually falls, in some cases, within a span of three to four weeks. We've seen that. But however, the challenge that comes is that sometimes when the chlorhexidine gel gets

mixed admixed with some fluids, it could form a nidus for infection. And at that point, we encourage mothers to change to the drops now. Yes.

3. Kindly describe the factors facilitating provision of chlorhexidine gel/solution

Okay. Some of the factors that facilitate the provision of the chlorhexidine is the fact that the hospital procures the chlorhexidine such that it's given to each and every mother after delivery. So I think the procurement part of it is a key factor for us. Thank you.

4. What Challenges do you experience with provision of chlorhexidine gel?

I will say that maybe not much of challenges but what I experience is that some mothers don't know how to keep well with their hygiene. And being that this is a gel and the hygiene is a bit poor, you find that the gel don't clear off, and it can form a nidus for infection when admixed with other fluids. I think, to me, that's a major challenge.

5. What would you suggest would lead to an increase in use of CHX?

I think what will lead to an increase in the use is basically health education for mothers through the NC care, such that all mothers first, and foremost, they deliver in the hospital. And when they deliver, they should be told the importance of cord care, even through the NC clinic. So when we empower them in the NC, that will lead to an increase in the use. And also those who even delivers at home, when they're empowered about cord care, they'll take initiative to come to the hospital for the chlorhexidine.

6. Comment on the IEC materials on CHX

I will say that the IEC materials are adequate. For instance, in my facility, they're in in the postnatal wards and also in the MCH clinics. And I think for that helps in the empowerment.

INDEX 23 – NAZARETH HOSPITAL

QUALITATIVE STUDY

A SEMI-STRUCTURED QUESTIONNAIRE

This questionnaire will be used to guide the interviewer.

Please let us record the following information that will be held confidentially and used only for the purpose of this research.

Bio data of the interviewee

Age…………………………… Professional line…………………….

Years of practice ……………..

Period of stay (years) in the specific station ……………………………

1. Please describe the existing cord care practices in our Kiambu County

We use chlorhexidine for cord care, and when it is not available, we use surgical spirit.

2. How would you describe your experience with CHX cord care antiseptic use in your hospital?

So the experience I have with chlorhexidine use, we have not had septic cords, and it's very easy to use.

3. Kindly describe the factors facilitating provision of chlorhexidine gel/solution

So we are using chlorhexidine because it is easy to use and it has good results.

4. What Challenges do you experience with provision of chlorhexidine gel?

Okay. So the challenges we have mainly is there is a time that it's not available.

5. What would you suggest would lead to an increase in use of CHX?

Yes. So the use of chlorhexidine can be increased by making it available to all at an affordable cost.

6. Comment on the IEC materials on CHX

So for us at Plainsview, chlorhexidine has to be given immediately after delivery, and the mothers are taught how to use it. So we do not have posters showing the issue of chlorhexidine because it's a must that every mother would get it after delivery.

INDEX 23 – NAZARETH HOSPITAL

QUALITATIVE STUDY

A SEMI-STRUCTURED QUESTIONNAIRE

This questionnaire will be used to guide the interviewer.

Please let us record the following information that will be held confidentially and used only for the purpose of this research.

Bio data of the interviewee

Age………………………… **Professional line…………………….**

Years of practice ………………

Period of stay (years) in the specific station ……………………………

1. Please describe the existing cord care practices in our Kiambu County

So we want to start recording. But allow me, I'm also trying to test. There are some tests I'm doing.

Interviewee: It's okay. It's okay. It's okay.

Interviewer: I'm sure.

2. How would you describe your experience with CHX cord care antiseptic use in your hospital?

In comparison to the mothers that usually come for the postnatal checkup, that is, one week post-delivery, those that have been using maybe the surgical spirit for cord care clinic compared

to those that have been using chlorhexidine, for the chlorhexidine, after the one week, the cord has already healed.

3. Kindly describe the factors facilitating provision of chlorhexidine gel/solution

Okay. In our facility, every postnatal mother, okay, post-delivery, before they are discharged [inaudible] each and every one is given the chlorhexidine. So they don't have to buy. It's usually in the hospital.

4. What Challenges do you experience with provision of chlorhexidine gel?

In the hospital ama?

Interviewer: Yes. In the hospital, I mean, with the mother.

Interviewee: Maybe at times, for example, few months ago, there was a time maybe they were out of stock, and we went to advice the mother to use surgical spirit. Yeah.

5. What would you suggest would lead to an increase in use of CHX?

I would say maybe it could be even not only here but in other different facilities, people do not have the knowledge on the use of chlorhexidine. So if they are sensitized on the importance of using it, then I think the uptake will go high.

6. Comment on the IEC materials on CHX

So we only give health messages to the mothers before they are discharged on how they are going to use it, that is, after discharge.

Interviewer: So do you have any here?

Interviewee: No.

INDEX 23 – NAZARETH HOSPITAL

QUALITATIVE STUDY

A SEMI-STRUCTURED QUESTIONNAIRE

This questionnaire will be used to guide the interviewer.

Please let us record the following information that will be held confidentially and used only for the purpose of this research.

Bio data of the interviewee

Age………………………… **Professional line…………………….**

Years of practice ……………..

Period of stay (years) in the specific station ……………………………

1. Please describe the existing cord care practices in our Kiambu County

The existing cord care are surgical spirit and chlorhexidine. But us in our facility, we use chlorhexidine and still some others usually come with telling us that they use the saliva and others that don't use anything.

2. How would you describe your experience with CHX cord care antiseptic use in your hospital?

Okay. The experience I have with chlorhexidine is very minimal because I don't work direct with the patient because the patient give the complaint to the doctors, and the doctors are the one who review the patient. So most of the induct information is with the doctor.

3. **Kindly describe the factors facilitating provision of chlorhexidine gel/solution**

Okay. The first factor is the cost and supply, and then the facility recommends the use of chlorhexidine.

4. **What Challenges do you experience with provision of chlorhexidine gel?**

So to my end, so far, I have no complaint concerning the use of chlorhexidine to the patient.

5. **What would you suggest would lead to an increase in use of CHX?**

One is educating the mothers on the use of chlorhexidine and then to have a constant supply of chlorhexidine and reduction of cost

6. **Comment on the IEC materials on CHX**

Okay. So far, we don't have such material, but I would rather recommend we have them so that the mother can learn or be taught more about the use of chlorhexidine.

INDEX 23 – NAZARETH HOSPITAL

QUALITATIVE STUDY

A SEMI-STRUCTURED QUESTIONNAIRE

This questionnaire will be used to guide the interviewer.

Please let us record the following information that will be held confidentially and used only for the purpose of this research.

Bio data of the interviewee

Age…………………………… Professional line…………………….

Years of practice ……………..

Period of stay (years) in the specific station ……………………………

1. Please describe the existing cord care practices in our Kiambu County

The cord care in Kiambu County that I'm aware of is the use of chlorhexidine and surgical spirit

2. How would you describe your experience with CHX cord care antiseptic use in your hospital?

Okay. The experience with the chlorhexidine with the postnatal mothers, it's effective simply because it's easier to use. Again, there are no infections that come with the mothers during the postnatal checkup.

3. Kindly describe the factors facilitating provision of chlorhexidine gel/solution

We recommend the use of chlorhexidine in our facility simply because it's cheap. Again, it's easy to use and it prevents the infections.

4. What Challenges do you experience with provision of chlorhexidine gel?

There are no challenges that come along with the use of chlorhexidine simply because it's easy to use and the mothers understands how to use it.

5. What would you suggest would lead to an increase in use of CHX?

The two factors that can increase the use of chlorhexidine, it's by lowering the cost to make it more available to all the mothers, and again, educate the mothers well about it during their antenatal clinics.

6. Comment on the IEC materials on CHX

For now, we don't have those materials, but they're important because they're easy to help when teaching the mother about the chlorhexidine. Again, it will become easy for the nurses who are around the ward. At least, they can teach one thing about the use of the chlorhexidine.

INDEX 23 – NAZARETH HOSPITAL

QUALITATIVE STUDY

A SEMI-STRUCTURED QUESTIONNAIRE

This questionnaire will be used to guide the interviewer.

Please let us record the following information that will be held confidentially and used only for the purpose of this research.

Bio data of the interviewee

Age………………………… **Professional line…………………….**

Years of practice ……………..

Period of stay (years) in the specific station ……………………………

1. Please describe the existing cord care practices in our Kiambu County

There is the use of surgical spirit cord care, saliva, especially for the traditional mothers

2. How would you describe your experience with CHX cord care antiseptic use in your hospital?

The effectiveness of cord care is better compared with other methods, like the surgical spirit or saliva for the traditional mothers because with the report of the mothers, it easier for them to use the cord care other than washing or cleaning the cord with the surgical spirit. Number two, in comparison with the mothers that have been using the surgical spirit and the cord care, during the postnatal checkup one week post-delivery, the mothers who have been using the cord care, the cords are healed faster compared with other methods.

3. Kindly describe the factors facilitating provision of chlorhexidine gel/solution

We are providing the cord care to the mothers to avoid the cord infections because earlier on when the mothers were using the surgical spirit because of maybe different hygiene measures, most of the babies were getting sepsis and cord infections, but while using the cord care, the number of sepsis or cord infections have reduced. So that's why we recommend using the cord care compared to surgical spirit.

4. What Challenges do you experience with provision of chlorhexidine gel?

We have not experienced any challenge because we provide the cord care to the mothers before the discharge.

5. What would you suggest would lead to an increase in use of CHX?

I would suggest that all mothers to be given [inaudible] ama to be encouraged to use it and then let there be constant supply of the cord care. Yes.

6. Comment on the IEC materials on CHX

We just provide the charts, especially on the postnatal ward, where the mothers are served just few hours of few hours post-delivery so that they can see the importance of using the cord care.

INDEX 23 – NAZARETH HOSPITAL

QUALITATIVE STUDY

A SEMI-STRUCTURED QUESTIONNAIRE

This questionnaire will be used to guide the interviewer.

Please let us record the following information that will be held confidentially and used only for the purpose of this research.

Bio data of the interviewee

Age………………………… **Professional line…………………….**

Years of practice ……………

Period of stay (years) in the specific station ……………………………

1. Please describe the existing cord care practices in our Kiambu County

The existing cord care practice in Kiambu are use of surgical spirit and chlorhexidine.

2. How would you describe your experience with CHX cord care antiseptic use in your hospital?

We recommend and encourage and teach the mothers on the use of chlorhexidine.

3. Kindly describe the factors facilitating provision of chlorhexidine gel/solution

Factors that facilitate us in using chlorhexidine is that it's readily available and easy to use.

4. What Challenges do you experience with provision of chlorhexidine gel?

The common challenge that we face is that mothers believe in the use of some traditional methods like use of saliva.

5. What would you suggest would lead to an increase in use of CHX?

To increase the use of chlorhexidine, we are supposed to create awareness for the mothers, reduce the cost, and maintain constant supply.

6. Comment on the IEC materials on CHX

So we currently do not have the IEC materials, but we recommend that they be made available.

INDEX 23 – NAZARETH HOSPITAL

QUALITATIVE STUDY

A SEMI-STRUCTURED QUESTIONNAIRE

This questionnaire will be used to guide the interviewer.

Please let us record the following information that will be held confidentially and used only for the purpose of this research.

Bio data of the interviewee

Age…………………………… Professional line…………………….

Years of practice ……………..

Period of stay (years) in the specific station ……………………………..

1. Please describe the existing cord care practices in our Kiambu County

Yeah. Of course, the-

Interviewer: I'm recording that, which is... It is still okay. If you allow me, it is still okay what I'm recording that, or I cancel that?

Interviewee: It's okay. Yeah. So I see most of the people still use... We used to use a lot of surgical spirit, okay, alcohol, yeah, maybe chlorhexidine came on. Yeah. It was used quite a bit, but I personally have not had any experience with chlorhexidine. So we finally settled for dry cord care, but we still get moms who come in with instructions to use alcohol and some come with chlorhexidine. But once they come to our unit, usually we advise them to use dry cord care.

2. How would you describe your experience with CHX cord care antiseptic use in your hospital?

Not use much of it. The only thing I saw is the cord, some take too long to fall off. Then number two, we had one, two babies who got contact dermatitis with it. Yeah. Otherwise, I have not had much experience with chlorhexidine. Yeah.

3. Kindly describe the factors facilitating provision of chlorhexidine gel/solution

Because I've told you I don't use chlorhexidine. It was brought here, but we don't use it in cleaning. [inaudible] we don't. We use dry cold care. Wash hands [inaudible]. Yeah.

4. What Challenges do you experience with provision of chlorhexidine gel?

I'm not using it. So I don't know if you mean by what challenges. We are not using it. It was procured by Kiambu, but yeah, the County knows we are not using it here in newborn unit.

Interviewer: Okay. What reasons would you say for not using?

Interviewee: World over, you go, it's dry cord care. Number two, since we stopped or we haven't used anything on those cord stamps, they heal very well, no complications.

5. What would you suggest would lead to an increase in use of CHX?

Personally, dry cord care is working for me. Even Ruiru called me, "What is this? The cords are not falling off?" I said, "Us, we don't apply anything on those cords, no chlorhexidine." If the cords dry, roll back the diaper to expose.

6. Comment on the IEC materials on CHX

Yeah. Even in maternity, postnatal, now they also... Maybe the one-one here and there. But most of them, it's dry cord care. Yeah.

Interviewer: *Okay.*

Interviewee: *Well, I don't know what you found, but-*

INDEX 23 – NAZARETH HOSPITAL

QUALITATIVE STUDY

A SEMI-STRUCTURED QUESTIONNAIRE

This questionnaire will be used to guide the interviewer.

Please let us record the following information that will be held confidentially and used only for the purpose of this research.

Bio data of the interviewee

Age…………………………… Professional line…………………….

Years of practice ……………..

Period of stay (years) in the specific station ……………………………

1. Please describe the existing cord care practices in our Kiambu County

So currently, in Kiambu County, there are different things that are being done. One, there' are some places and institutions that are using chlorhexidine for cord care. Then a number of institutions are using surgical spirit. There are some people who even believe on doing nothing to cord care. There are people who are using normal saline for cord care.

2. How would you describe your experience with CHX cord care antiseptic use in your hospital?

So in Gatundu, we used chlorhexidine for quite some time, 2019, 2020, on all our newborns. But with time, our use of chlorhexidine has gone down because of complaints that were coming so much from the mothers. There was delayed cord separation. We had even cases of babies going up to 35 days without the cord having fallen off, increased risk of sepsis because of delay in

starting the chlorhexidine. Chlorhexidine initially we realized initially It was not being applied immediately after birth. So that was contributing to the increase of sepsis on the cord. So currently, our uptake in the hospital in Gatundu Level 5 of chlorhexidine is very low.

3. Kindly describe the factors facilitating provision of chlorhexidine gel/solution

Currently in our hospital, we are not using chlorhexidine, and even if we have it, we're not using it. We are using surgical spirit in all our newborn papers because of now the complaints that were coming up, the delayed cord separation and increased risk of cord sepsis.

4. What Challenges do you experience with provision of chlorhexidine gel?

So in Gatundu currently, the demand of chlorhexidine is not there because what I've discussed earlier, we do not like how our patients were... the outcomes we were having in our babies. So we stopped using chlorhexidine.

Interviewer: Is the staff trained?

Interviewee: Yes, yes, yes. The staff are trained. They have been trained. They even had CMEs on chlorhexidine, by the way. A lot of trainings. Yeah. We had many trainings on chlorhexidine. And we were all very conversant with the use of chlorhexidine.

5. What would you suggest would lead to an increase in use of CHX?

Into the use of chlorhexidine. I think we should retrain staff, and we should do away that negative. We saw it is not working for some mothers. We were very fast to stop it. Maybe we should see whether we can have an attitude change, which can be brought by continuous training. We see whether probably there was a wrong news, whether the mothers were going home were using it properly. So I think we can retrain the healthcare workers and retrain the mothers.

6. Comment on the IEC materials on CHX

Yes, we have them. We have IEC materials in our labor ward and even in our newborn unit and in the postnatal wards. They're all over in the wards. So the information is there.

Interviewer: *And they're adequate in information?*

Interviewee: *Yes, yes, yes. The information that is there is adequate.*

INDEX 23 – NAZARETH HOSPITAL

QUALITATIVE STUDY

A SEMI-STRUCTURED QUESTIONNAIRE

This questionnaire will be used to guide the interviewer.

Please let us record the following information that will be held confidentially and used only for the purpose of this research.

Bio data of the interviewee

Age…………………………… Professional line…………………….

Years of practice ……………..

Period of stay (years) in the specific station ……………………………

1. Please describe the existing cord care practices in our Kiambu County

So here in Thika Level 5, Kiambu County, we usually use chlorhexidine. But the mothers are given in postnatal ward after delivery, and they are advised to continue at home until two weeks. So when they come back after two weeks, we are able to assess and then we know if the cord has healed or not yet.

2. How would you describe your experience with CHX cord care antiseptic use in your hospital?

When they are given the chlorhexidine it works. But at times, always there is always stock-ons. So when we tell the mother to go and buy the chlorhexidine, at times they're not able to buy. So they buy the spirit at 50 bob, and they can deal with it.

3. Kindly describe the factors facilitating provision of chlorhexidine gel/solution

So because they don't dry up, they're supposed to dry and this and not sepsis. Yeah.

Interviewee: Anything else?

Interviewer: So when there is lack, there is a lot of admissions. Yeah. And this causes even sepsis to these babies [inaudible] sometimes even duties.

4. What Challenges do you experience with provision of chlorhexidine gel?

The chlorhexidine. And sometimes the mothers, they don't know how to use the chlorhexidine. Yes.

Interviewer: So what do you think should be done?

Interviewee: There should be a supply consistently. And then when there is the chlorhexidine in the postnatal ward, they are shown how to clean the cord that continues even at home.

5. What would you suggest would lead to an increase in use of CHX?

We should have constant supply of the chlorhexidine. So when we have it, it becomes a habit and a practice at the Thika Level 5 so that the mothers will switch on from spirit and we adapt chlorhexidine. It becomes a routine.

6. Comment on the IEC materials on CHX

Antenatal clinic, we don't have them. I don't know about postnatal clinic. But in the antenatal, we don't have them. We can also introduce it in our microteaching in the morning so that the mothers are [inaudible]. Those who are bringing the sick babies, those who are coming for the antenatal clinic, those who are coming for immunization services, everybody knows there is something else that is coming up which is very good for these babies called chlorhexidine, and then we have the posters. Yes.

Interviewer: *For now, the posters that are there, are they giving adequate information or the posters that you have seen?*

Interviewee: *Yes. It's adequate, but they should distribute them.*

INDEX 23 – NAZARETH HOSPITAL

QUALITATIVE STUDY

A SEMI-STRUCTURED QUESTIONNAIRE

This questionnaire will be used to guide the interviewer.

Please let us record the following information that will be held confidentially and used only for the purpose of this research.

Bio data of the interviewee

Age………………………… **Professional line…………………….**

Years of practice ……………..

Period of stay (years) in the specific station ……………………………

1. Please describe the existing cord care practices in our Kiambu County

Currently, the mothers are being advised to use the surgical spirit in cleaning of the cord right after birth all the way up to three weeks post-delivery. That is the current practice that is there.

2. How would you describe your experience with CHX cord care antiseptic use in your hospital?

It used to be there. Some years back, we used to have the chlorhexidine. And the mothers were a bit rigid. They are used to utilizing the surgical spirit and some say they do not apply anything. But during my practice, I saw it was far much better in terms of the cord care unlike utilization of the surgical spirit.

3. Kindly describe the factors facilitating provision of chlorhexidine gel/solution

When you mean provision, is it its availability? The gel was more preferred by the mothers due to its small packaging. Also, the packaging was also attractive. It was easy to carry back home, and it was easy to apply. Yeah.

4. What Challenges do you experience with provision of chlorhexidine gel?

Yes. The shortcoming of now the chlorhexidine solution or the gel was the availability, the sustainability of its availability to the facilities and also clear advise on the mothers on the uptake. Some mothers used to raise eyebrows, "Why are we using it instead of these other methods?" So it was good at that time mothers to be explained to the advantages of it and also now the shortcomings in terms of the sustainability of its provision.

5. What would you suggest would lead to an increase in use of CHX?

Good or elaborative health education to both the health workers and also to the clients on the usage and the advantages of now the chlorhexidine gel or the solution and also sustainable supply of the chlorhexidine gel.

6. Comment on the IEC materials on CHX

That time?

Interviewer: Even now.

Interviewee: No. I see materials are available currently.

Interviewer: Have you ever had any?

Interviewee: None.

Interviewer: You have not had any AIC materials.

Interviewee: No. No.

Interviewer: *Which then means you cannot describe them since you haven’t seen any.*

Interviewee: *Yes. Yes.*

INDEX 23 – NAZARETH HOSPITAL

QUALITATIVE STUDY

A SEMI-STRUCTURED QUESTIONNAIRE

This questionnaire will be used to guide the interviewer.

Please let us record the following information that will be held confidentially and used only for the purpose of this research.

Bio data of the interviewee

Age…………………………… Professional line…………………….

Years of practice ……………..

Period of stay (years) in the specific station ……………………………

1. Please describe the existing cord care practices in our Kiambu County

What we usually use is surgical spirit. Previously, we used to use chlorhexidine. Because of the misunderstanding on how it's supposed to be used, the mothers were advised to be using surgical spirit on the cord of their babies.

Interviewer: Is that all?

Interviewee: There are other practices I've heard being used in other facilities in Kiambu County, like just using clean water instead of using surgical spirit, just clean water, cleaning the cord with cotton wool and cleaning it.

2. How would you describe your experience with CHX cord care antiseptic use in your hospital?

The experience has not been positive because of the feedback that we got from mothers or from the nurses who attend to those mothers who come back with the sick babies because of misunderstanding the use of chlorhexidine. Yeah.

3. Kindly describe the factors facilitating provision of chlorhexidine gel/solution

Cost of the chlorhexidine has to be friendly and how it is being marketed also to the healthcare workers. It depends. If they take it up positively, they understand how it's supposed to be used, the message will be forwarded to the mothers positively. Yeah.

4. What Challenges do you experience with provision of chlorhexidine gel?

Challenges with chlorhexidine gel. They're still the same. Understanding how it's supposed to be used is a challenge, and also cost can be a challenge to some of the patients. And the cost also can be a determining factor by the facility, the pharmacist that is supposed to order the drug. Yeah, basically cost and understanding how to use it.

5. What would you suggest would lead to an increase in use of CHX?

So number one is cost. Cost has to be friendly to the consumer. Number two, the packaging. Maybe some packing it in such a way that does not resemble another formulation that a patient can mistake, a layman can mistake the use of that drug. The other one is educating, first of all, the healthcare worker on how to use it so that the healthcare worker is empowered to also educate the mothers on how to use it. And yeah, basically that.

6. Comment on the IEC materials on CHX

I have not seen. Basically, I have not come across any materials. And maybe it's because of where it's supposed to be where a healthcare worker is stationed. It's mostly is supposed to be where maternal and child health areas. Maybe we should make the materials available in other

departments that are not necessarily maternal and child health related for basically general knowledge. Yeah.

Interviewer: *Have you participated in any of the trainings on use of the chlorhexidine?*

Interviewee: *No.*

Interviewer: *So you have not seen any in the posters?*

Interviewee: *No.*

INDEX 23 – NAZARETH HOSPITAL

QUALITATIVE STUDY

A SEMI-STRUCTURED QUESTIONNAIRE

This questionnaire will be used to guide the interviewer.

Please let us record the following information that will be held confidentially and used only for the purpose of this research.

Bio data of the interviewee

Age…………………………… Professional line…………………….

Years of practice ……………..

Period of stay (years) in the specific station ……………………………

1. Please describe the existing cord care practices in our Kiambu County

So the current cord care practice in Thika Level 5 I would say, predominantly because that's where I practice is dry cord care. I hardly do surgical spirit or chlorhexidine or any other medication directly with the cord. So they leave the cords to dry and fall off from their own. And occasionally, when we get kids with septic cords, we get them… Some are doing surgical spirit. A few cases we get with chlorhexidine. Most of the time they're referrals in from peripheral facilities. Particularly, the few we would get from Murang'a would come with chlorhexidine. But what I see currently is hardly are we seeing chlorhexidine being used in babies for cord care.

2. How would you describe your experience with CHX cord care antiseptic use in your hospital?

Using chlorhexidine.

Interviewer: *It's okay, please.*

Interviewee: *Initially, when we started using chlorhexidine, of course the availability was good and a lot of mothers were taking it and embracing it, of course, with the initial challenge of having the gel and the one that looked like drops, the ones that you drop on the cord. But then with those eye accidents with the drops, the drops were withdrawn, and most of the time, we would get the gel. Now, the problem with the gel was that either it was too thick and it kept leaving the cord moist, and so it would take longer for the cord to drop off. And so with that challenge, and of course every mother wants their cord to drop within a few weeks of administration of whatever it is they're using for cord care. So a lot of children would come with delayed cord dropping. And so they would now end up having to come for reviews to get seen by the pediatrician. Of course, it disturbs them that three, four weeks down the line, the cord has not dropped. And in my own assessment, I thought probably it was because chlorhexidine fluoxetine is more of a lotion, a soap rather than an alcohol and doesn't dry the cord as fast. But in most of those cases, just cessation of use allowed the cord to dry, and it fell off with time. So in our practice, we did not particularly switch from, let's say, chlorhexidine to spirit. We just did the chlorhexidine, and that was it, initially. But then, at some point, stockouts. We could not even see the chlorhexidine. And so we went back to our normal life. And I think pharmacy, when they brought it back and they were ordering, we were not using it because we were seeing our cords were drying better, and we were doing okay without it. So that's how we lost the bus.*

3. Kindly describe the factors facilitating provision of chlorhexidine gel/solution

So the erratic supplies by the county has allowed us to explore other alternatives of cord care. And for us, leaving the cord to dry on its own has worked very well. Okay. And so when now it comes to ordering, with the decreased efficacy, so the demand also goes down. So we have skinny budgets in our facilities. Chlorhexidine is not one of the drugs we would prioritize to buy. If you were to ask us what are the things we would want to buy for your facility, it will not be chlorhexidine for cord care. Yeah. We would probably be buying saline nasal drops for nasal decongestion than buying chlorhexidine. So those challenges about efficacy were there. And then the erratic supplies for the county, particularly because of those financial constraints the counties have been having have made it easy for us to try out other things because in the absence of chlorhexidine, what else was being done to allow cords to heal. And that's how we got here. So efficacy, erratic supplies, what else? Yeah, I think that's it.

4. What Challenges do you experience with provision of chlorhexidine gel?

Initially, when the drops and gel were available, it was harder to find gel than to find the drops. The drops are more readily available. But then with the eye accidents related to application of the drops in the eyes, chlorhexidine gel was supposed to be made much more available to the clients. But now, yeah, the supplies and the county, it's quite irregular. The stock-outs are very, very, very common because of the budgetary constraints in the county. But yeah, I think that answers that question. So readily availability of the chlorhexidine gel. Yeah. It would be the ideal at that point. But clearly because of not having it readily available, we found options.

5. What would you suggest would lead to an increase in use of CHX?

That's a tricky question, in the sense that other options have worked well. So we are not under pressure to get back chlorhexidine on board. But it is not because there was a knowledge gap. I don't think it was a knowledge gap that made chlorhexidine not popular. The knowledge was there because the time, everybody switched to chlorhexidine. The knowledge was there, and the times it was available, they will all order for the patients. The chlorhexidine would come together with the identification tag, the [inaudible] and the chlorhexidine for the cord care at the time, you're going to deliver any mother. That was the standard practice. I think what made it unpopular is the delays in cord drop. Yes. Lakini the question is asking, what can be done to increase? I don't think there's a knowledge gap. I don't think there's a knowledge gap. I don't think I've answered that question.

Interviewer: What do you think can be done to improve?

Interviewee: To improve chlorhexidine use? I'm not sure it would be taken up. Yeah. To be very sincere. Yeah.

6. Comment on the IEC materials on CHX

They're available. The AIC material is available in the ward as tea cups. And yeah, that's the fight because-

Interviewer: In the maternity ward, that's where they are?

Interviewee: In the newborn unit. There's a notice board in the newborn unit. Pediatrics doesn't have.

INDEX 23 – NAZARETH HOSPITAL

QUALITATIVE STUDY

A SEMI-STRUCTURED QUESTIONNAIRE

This questionnaire will be used to guide the interviewer.

Please let us record the following information that will be held confidentially and used only for the purpose of this research.

Bio data of the interviewee

Age………………………… **Professional line…………………….**

Years of practice ……………

Period of stay (years) in the specific station ……………………………

1. Please describe the existing cord care practices in our Kiambu County

Okay. Here in our health facility, we do not use the chlorhexidine gel because we realized even when you don't even use anything, as long as you educate the mother on hygiene, the cords do not become septic. So we realized there was... We discussed with our pediatrician and we just decided to leave the cord just like that. Don't put anything, don't put water, don't put the spirit, and don't put chlorhexidine, as long as you keep it clean. And we have not had any case of sepsis.

2. How would you describe your experience with CHX cord care antiseptic use in your hospital?

I cannot comment on that because we have not been using the chlorhexidine gel. So for that one, I cannot comment more.

Interviewer: *Do you know anything about it?*

Interviewee: *Yeah, we know. We know about it.*

3. Kindly describe the factors facilitating provision of chlorhexidine gel/solution

I think about the contributing factors, I think one thing that we need maybe to revisit the issue of the... need to do a CME on it once again, because it is very controversial. Some, they say you need... The guys from the Afya House when they come, they say, "No, you must use it. You must use it." But then we are agreed for us, we don't use it here, we have not been using it, and we have not had any cases of neonatal sepsis. So I think we need to maybe have a sitting and do a CME, and now we have to weigh the pros, the ups and downs, whether we need to use it or not.

4. What Challenges do you experience with provision of chlorhexidine gel?

I don't think provision can be an issue because even if you were to say we are going to be using it, I don't think we'd have an issue of it being not available. I think provision will be there. It's only that we don't use it because like I said, we realized we don't have any case of neonatal sepsis even if we do not use it. So I don't think provision will be an issue.

5. What would you suggest would lead to an increase in use of CHX?

I think the only thing we need to do is just to have CMEs so that... CMEs, CMEs. That is the only thing we need to, the only need we need.

6. Comment on the IEC materials on CHX

We had a few before we moved on from the old building to here. But for now, we don't have here. We don't have anything. We don't have about chlorhexidine. We don't have.

Interviewer: *Why did you come with them, the ones that you had?*

Interviewee: *I don't know who had... They were provided, I think by... Was it the government? But someone brought them. We were provided with some. But during that time of moving, I think now they got misplaced somehow. But for now, we don't have.*

Interviewer: *Were they useful when you had them?*

Interviewee: *Yeah, they were, they were.*

Interviewer: *So you cannot say they have any deficiency that you noted?*

Interviewee: *No, no, no.*

Hospital	Age	Cadre	Practice years	_index
nazareth	56	midwife		1
githunguri_level_3	23	midwife	0.5	2
ruiru	34	pharmacist	6	3
ruiru	34	antenatal_nurse	7	4
ruiru	27	post_natal_nurse	5	5
ruiru	28	midwife	2	6
ruiru	54	paediatrician_medical_officer	28	7
ruiru	27	post_natal_nurse		8
thika_level_5	54	antenatal_nurse	30	9
thika_level_5	35	post_natal_nurse	11	10
thika_level_5	42	pharmacist	13	11
thika_level_5	44	paediatrician_medical_officer	5	12
thika_level_5	36	midwife	13	13
thika_level_5	57	paediatrician_medical_officer	21	14
kihara_level_4	35	post_natal_nurse	10	15
kihara_level_4	42	midwife	7	16
kihara_level_4	30	antenatal_nurse	6	17
kihara_level_4	28	pharmacist	5	18
kihara_level_4	33	paediatrician_medical_officer	7	19
kiambu_level_5	30	pharmacist	7	20
kiambu_level_5	28	midwife	4	21
kiambu_level_5	31	midwife	7	22
nazareth	38	antenatal_nurse	10	23
kihara_level_4	34	pharmacist	12	24
nazareth	35	pharmacist	8	25
nazareth	27	paediatrician_medical_officer	1	26
nazareth	33	midwife	10	27
nazareth	26	paediatrician_medical_officer	1	28
githunguri_level_3	27	post_natal_nurse	4	29
githunguri_level_3	37	midwife	12	30
githunguri_level_3	31	paediatrician_medical_officer	7	31
githunguri_level_3	38	pharmacist	10	32
githunguri_level_3	36	antenatal_nurse	11	33
kiambu_level_5	33	pharmacist	7	34
kiambu_level_5	36	midwife	12	35
kiambu_level_5	37	paediatrician_medical_officer	12	36
thika_level_5	40	paediatrician_medical_officer	15	37
kiambu_level_5	36	paediatrician_medical_officer	12	38
