# Supplementary material for: “...In Kiambu county, there are different things being done”: A qualitative exploration of healthcare workers’ experiences with cord care practices
Source: PLoS One. 2025 Jun 18;20(6):e0326506. doi: 10.1371/journal.pone.0326506 (PMC12176292; doi:10.1371/journal.pone.0326506)
Supplement: Attachment — Submitted filename: PONE-D-25-16839_reviewer_GB.pdf [file pone.0326506.s003.pdf]

PLOS ONE

“...in Kiambu county, there are different things being done”: A Qualitative Study Exploring the Experiences of Healthcare Workers with 7.1% Chlorhexidine digluconate Cord Care in Kiambu County, Kenya

--Manuscript Draft--

**Manuscript Number:**	PONE-D-25-16839
**Article Type:**	Research Article
**Full Title:**	“...in Kiambu county, there are different things being done”: A Qualitative Study Exploring the Experiences of Healthcare Workers with 7.1% Chlorhexidine digluconate Cord Care in Kiambu County, Kenya
**Short Title:**	The Experiences of Healthcare Workers with Cord Care in Kiambu County, Kenya
**Corresponding Author:**	Prabhjot Kaur Juttla, BSc<br>University of Nairobi College of Health Sciences: University of Nairobi Faculty of Health Sciences<br>Nairobi, KENYA
**Keywords:**	CHX uptake; Kiambu county; Cord-care; Neonatal sepsis; Experiences
**Abstract:**	Introduction<br><br>Neonatal infections remain a major cause of morbidity and mortality in low-resource settings, necessitating effective interventions for umbilical cord care. The World Health Organization (WHO) recommends the use of 7.1% chlorhexidine (CHX) solution for cord care in settings with high neonatal mortality. However, its adoption varies across different regions. This study explored healthcare workers' experiences with CHX use for umbilical cord care in Kiambu County, Kenya, a low-income area with high hospital birth rates.<br><br>Methods<br><br>This study employed an exploratory qualitative design. In-depth interviews (IDIs) were conducted with 38 healthcare providers between October 6th and November 9th, 2022, in various public healthcare facilities in Kiambu County. Participants were selected based on their experience with neonatal care and cord management practices. Interviews focused on their perceptions, experiences, and challenges regarding CHX use. Data were analyzed thematically to identify key factors influencing CHX adoption.<br><br>Results<br><br>The uptake of CHX in public health facilities in Kiambu County was low. Availability and cost were significant barriers to its consistent use. While some healthcare providers acknowledged CHX as effective, easy to use, and beneficial in reducing infections, others considered it expensive and unnecessary. The study identified multiple factors influencing CHX adoption, including availability, affordability, accessibility, perceived effectiveness, high demand, clinical outcomes, reduced infections, and government support. Many providers noted that mothers often preferred traditional cord care methods or other antiseptics over CHX.<br><br>Conclusion<br><br>To improve CHX uptake, targeted advocacy and training initiatives for healthcare providers and mothers are essential. Additionally, reassessing CHX supply chain management and cost-related barriers at the county level may help enhance its accessibility. Further research is needed to evaluate regional variations in CHX utilization and identify strategies to optimize neonatal cord care practices across Kenya.
**Order of Authors:**	James Maina Githinji
	Angeline Chepchirchir

Powered by Editorial Manager® and ProduXion Manager® from Aries Systems Corporation

	Brian Ombura Nyanchoka
	Prabhjot Kaur Juttla, BSc
	Ruth Nduati
**Additional Information:**	
**Question**	**Response**
**Financial Disclosure**<br><br>Enter a financial disclosure statement that describes the sources of funding for the work included in this submission. Review the submission guidelines for detailed requirements. View published research articles from *PLOS ONE* for specific examples.<br><br>This statement is required for submission and **will appear in the published article** if the submission is accepted. Please make sure it is accurate.<br><br>**Funded studies**<br>Enter a statement with the following details:<br>• Initials of the authors who received each award<br>• Grant numbers awarded to each author<br>• The full name of each funder<br>• URL of each funder website<br>• Did the sponsors or funders play any role in the study design, data collection and analysis, decision to publish, or preparation of the manuscript?<br><br>Did you receive funding for this work?	Yes
Please add funding details.<br>as follow-up to "**Financial Disclosure**<br><br>Enter a financial disclosure statement that describes the sources of funding for the work included in this submission. Review the submission guidelines for detailed requirements. View published research articles from *PLOS ONE* for specific examples.<br><br>This statement is required for submission and **will appear in the published article** if	This study was funded by NIH Fogarty Grant R 25TW011212 awarded to Prof. Ruth Nduati. The opinions and conclusions are by the authors and do not necessarily represent the funding institution position.

the submission is accepted. Please make sure it is accurate.<br><br>**Funded studies**<br>Enter a statement with the following details:<br>• Initials of the authors who received each award<br>• Grant numbers awarded to each author<br>• The full name of each funder<br>• URL of each funder website<br>• Did the sponsors or funders play any role in the study design, data collection and analysis, decision to publish, or preparation of the manuscript?<br><br>Did you receive funding for this work?"	
Please select the country of your main research funder (please select carefully as in some cases this is used in fee calculation).<br>as follow-up to "**Financial Disclosure**<br><br>Enter a financial disclosure statement that describes the sources of funding for the work included in this submission. Review the submission guidelines for detailed requirements. View published research articles from *PLOS ONE* for specific examples.<br><br>This statement is required for submission and **will appear in the published article** if the submission is accepted. Please make sure it is accurate.<br><br>**Funded studies**<br>Enter a statement with the following details:<br>• Initials of the authors who received each award<br>• Grant numbers awarded to each author<br>• The full name of each funder<br>• URL of each funder website<br>• Did the sponsors or funders play any role in the study design, data collection and analysis, decision to publish, or preparation of the manuscript?	KENYA - KE

Powered by Editorial Manager® and ProduXion Manager® from Aries Systems Corporation

Did you receive funding for this work?"	
**Competing Interests**<br><br>Use the instructions below to enter a competing interest statement for this submission. On behalf of all authors, disclose any competing interests that could be perceived to bias this work—acknowledging all financial support and any other relevant financial or non-financial competing interests.<br><br>This statement is required for submission and **will appear in the published article** if the submission is accepted. Please make sure it is accurate and that any funding sources listed in your Funding Information later in the submission form are also declared in your Financial Disclosure statement.<br><br>View published research articles from *PLOS ONE* for specific examples.<br><br>**NO authors have competing interests**<br><br>Enter: *The authors have declared that no competing interests exist.*<br><br>**Authors with competing interests**<br><br>Enter competing interest details beginning with this statement:<br><br>*I have read the journal's policy and the authors of this manuscript have the following competing interests: [insert competing interests here]*<br><br>* typeset	The authors have declared that no competing interests exist.
**Ethics Statement**<br><br>Enter an ethics statement for this submission. This statement is required if the study involved:<br><br>• Human participants<br>• Human specimens or tissue	Approval was obtained at various administrative levels, including county and sub-county authorities, before the study commenced. Ethical clearance was secured from the University of Nairobi/Kenyatta National Hospital (KNH) Ethical Review Committee (ERC) [P470/05/2022], County Government of Kiambu Department of Health Services, Kiambu County [KIAMBU/HRDU/22/09/14/RA_GITHINJI], and a research license obtained from the National Commission for Science, Technology and Innovation [NACOSTI/P/22/20446]. The participating facilities also provided permission to conduct the study in their facilities.

• Vertebrate animals or cephalopods<br>• Vertebrate embryos or tissues<br>• Field research<br><br>Write "N/A" if the submission does not require an ethics statement.<br><br>General guidance is provided below. Consult the submission guidelines for detailed instructions. **Make sure that all information entered here is included in the Methods section of the manuscript.**	

Powered by Editorial Manager® and ProduXion Manager® from Aries Systems Corporation

**Format for specific study types**<br><br>**Human Subject Research (involving human participants and/or tissue)**<br>• Give the name of the institutional review board or ethics committee that approved the study<br>• Include the approval number and/or a statement indicating approval of this research<br>• Indicate the form of consent obtained (written/oral) or the reason that consent was not obtained (e.g. the data were analyzed anonymously)<br><br>**Animal Research (involving vertebrate animals, embryos or tissues)**<br>• Provide the name of the Institutional Animal Care and Use Committee (IACUC) or other relevant ethics board that reviewed the study protocol, and indicate whether they approved this research or granted a formal waiver of ethical approval<br>• Include an approval number if one was obtained<br>• If the study involved *non-human primates*, add *additional details* about animal welfare and steps taken to ameliorate suffering<br>• If anesthesia, euthanasia, or any kind of animal sacrifice is part of the study, include briefly which substances and/or methods were applied<br><br>**Field Research**<br><br>Include the following details if this study involves the collection of plant, animal, or other materials from a natural setting:<br>• Field permit number<br>• Name of the institution or relevant body that granted permission	
**Data Availability**<br><br>Authors are required to make all data underlying the findings described fully available, without restriction, and from the time of publication. PLOS allows rare exceptions to address legal and ethical concerns. See the PLOS Data Policy and FAQ for detailed information.	Yes - all data are fully available without restriction

A Data Availability Statement describing where the data can be found is required at submission. Your answers to this question constitute the Data Availability Statement and **will be published in the article**, if accepted.<br><br>**Important:** Stating 'data available on request from the author' is not sufficient. If your data are only available upon request, select 'No' for the first question and explain your exceptional situation in the text box.<br><br>Do the authors confirm that all data underlying the findings described in their manuscript are fully available without restriction?	
**Describe where the data may be found in full sentences. If you are copying our sample text, replace any instances of XXX with the appropriate details.**<br><br>• If the data are **held or will be held in a public repository**, include URLs, accession numbers or DOIs. If this information will only be available after acceptance, indicate this by ticking the box below. For example: *All XXX files are available from the XXX database (accession number(s) XXX, XXX.).*<br>• If the data are all contained **within the manuscript and/or Supporting Information files**, enter the following: *All relevant data are within the manuscript and its Supporting Information files.*<br>• If neither of these applies but you are able to provide **details of access elsewhere**, with or without limitations, please do so. For example:<br><br>*Data cannot be shared publicly because of [XXX]. Data are available from the XXX Institutional Data Access / Ethics Committee (contact via XXX) for researchers who meet the criteria for access to confidential data.*<br><br>*The data underlying the results presented in the study are available from (include the name of the third party*	All relevant data are within the manuscript and its Supporting Information files.

and contact information or URL).<br>• This text is appropriate if the data are owned by a third party and authors do not have permission to share the data.<br><br>* typeset	
Additional data availability information:	

Powered by Editorial Manager® and ProduXion Manager® from Aries Systems Corporation

Manuscript

Click here to access/download;Manuscript;Manuscript 27_03_2025.docx

"...in Kiambu county, there are different things being done": A Qualitative Study Exploring the Experiences of Healthcare Workers with 7.1% Chlorhexidine digluconate Cord Care in Kiambu County, Kenya

Authors

James Maina Githinji[1,2], Angeline Chepchirchir [3], Brian Ombura Nyanchoka[4], Prabhjot Kaur Juttla[5*], Ruth Nduati [2]

Institutional affiliation:

[1] Department of Health, County Government of Kiambu, Kiambu County, Kenya

[2] Department of Paediatrics and Child Health, Faculty of Health Sciences, University of Nairobi, Nairobi, Kenya

[3] Department of Nursing, Faculty of Health Sciences, University of Nairobi, Nairobi, Kenya

[4] Department of Pharmacy, Jomo Kenyatta University of Agriculture and Technology, Nairobi, Kenya

[5] School of Medicine, Faculty of Health Sciences, University of Nairobi, Nairobi, Kenya

***Corresponding Author**

pkjuttla13@gmail.com (PKJ)

Abstract

Introduction: Neonatal infections remain a major cause of morbidity and mortality in low-resource settings, necessitating effective interventions for umbilical cord care. The World Health Organization (WHO) recommends the use of 7.1% chlorhexidine (CHX) solution for cord care in settings with high neonatal mortality. However, its adoption varies across different regions. This study explored healthcare workers' experiences with CHX use for umbilical cord care in Kiambu County, Kenya, a low-income area with high hospital birth rates.

Methods: This study employed an exploratory qualitative design. In-depth interviews (IDIs) were conducted with 38 healthcare providers between October 6th and November 9th, 2022, in various public healthcare facilities in Kiambu County. Participants were selected based on their experience with neonatal care and cord management practices. Interviews focused on their perceptions, experiences, and challenges regarding CHX use. Data were analyzed thematically to identify key factors influencing CHX adoption.

Results: The uptake of CHX in public health facilities in Kiambu County was low. Availability and cost were significant barriers to its consistent use. While some healthcare providers acknowledged CHX as effective, easy to use, and beneficial in reducing infections, others considered it expensive and unnecessary. The study identified multiple factors influencing CHX adoption, including availability, affordability, accessibility, perceived effectiveness, high demand, clinical outcomes, reduced infections, and government support. Many providers noted that mothers often preferred traditional cord care methods or other antiseptics over CHX.

Conclusion: To improve CHX uptake, targeted advocacy and training initiatives for healthcare providers and mothers are essential. Additionally, reassessing CHX supply chain management and cost-related barriers at the county level may help enhance its accessibility. Further research is

needed to evaluate regional variations in CHX utilization and identify strategies to optimize neonatal cord care practices across Kenya.

Key Words: CHX uptake; Kiambu county; Cord-care; Neonatal sepsis; Experiences

Background

Neonatal sepsis (NNS) is a significant contributor to neonatal mortality during the first month of life [1]. One of the main contributors to NNS is the development of omphalitis, which is the infection of the umbilical stump [2]. This infection often arises as a result of improper or inadequate umbilical cord care practices and is typically considered a medical emergency due to its rapid progression to NNS, which can ultimately lead to neonatal mortality in up to 15% of cases [3]. While this is relatively uncommon in developed nations, it is a significant contributor to neonatal mortality in developing nations [3]. Indeed, studies from Kenya and other East African countries have explored a range of substances used by mothers for umbilical cord care, including methylated spirits, hot water, saliva, herbal remedies, and even non-traditional items like red sand, ash, and toothpaste [4–9].

Due to this significant burden of NNS secondary to omphalitis, the World Health Organization (WHO) currently has a context-specific recommendation for newborns in settings where harmful traditional substances are prevalent and the neonatal mortality rate (NMR) exceeds 30 in 1000 live births [10]. This is a transition from the previous recommendation of surgical spirit [11], a recommendation which was also reflected in Kenya's 2009 National Guidelines for Quality

Perinatal Care [12]. However, Kenya adopted the new WHO guidelines nation-wide where all newborns should receive CHX for cord care [13].

However, conflicting evidence has arisen from large trials in Bangladesh and Zambia regarding the potential impact of the CHX protocol on infant mortality because it's use for a week has been associated with delayed cord detachment [14,15]. In the Zambian study, CHX cord applications did not seem to provide clear benefits for newborn babies in settings with predominantly facility-based deliveries and lower NMRs [15]. Sazawal *et al.* questioned the recommendation of CHX for reducing infant mortality in East Africa based on limited evidence from a study involving 47,545 births [16]. Another contrary finding in the African setting includes a study by Shwe *et al.* found that methylated spirit and 4% CHX gel had similar efficacy in treating umbilical stumps [17].

In Kiambu County, Kenya, the adoption of CHX for cord care has yet to be thoroughly evaluated from the perspective of healthcare workers (HCWs). As Kenya seeks to increase the uptake of CHX treatment to over 80% by 2026 [18], and given that CHX uptake in Kiambu county currently stands at 40.1% [19], evaluating the perspectives of HCWs will be crucial in achieving this goal. Understanding HCW experiences with CHX is essential for shaping health policy, especially in light of the high prevalence of harmful cord care practices in Kenya. This study aimed to assess HCWs' experiences with CHX, particularly in public health facilities, to identify the factors influencing its adoption.

Methods

Study Setting

This study was carried out in Kiambu County, Kenya, which has a population of over 2.4 million people. The County location coordinates are 1^0 10'S $36^0$50'E, and it neighbors six other counties, namely Nairobi City and Kajiado Counties to the South, Machakos to the East, Murang'a to the North East, Nyandarua to the North west, and Nakuru County to the West [20]. The county is 60% urban and 40% rural due to its proximity to the rapidly expanding Nairobi City.

The county has 505 health facilities, including 108 public, 64 faith-based, and 333 privately owned facilities. The study sought to gather the views and experiences of healthcare providers regarding the use of chlorhexidine (CHX) in Level 5, Level 4, and Level 3 public facilities, as well as private maternity hospitals and faith-based hospitals. One of the level 5 hospitals included in this study doubles as a teaching hospital for medical and nursing students.

The National Health Insurance Fund (NHIF), now defunct but was active during the period of the study, had a program called *Linda Mama* that covered the costs of antenatal care (ANC), delivery, and postnatal care (PNC) for all pregnant women [21]. However, it notably did not include the cost of chlorhexidine (CHX).

Study Population and Sampling

The study population consisted of over 2,600 HCWs providing health services in Kiambu County during the study period (6th October 2022 to 9th November 2022). No formal sample size was calculated and we applied the information saturation principle [22].

Study Design

The study adopted an exploratory qualitative design using one-on-one in-depth interviews (IDIs) guided by semi-structured question guides, conducted between 6th October 2022 to 9th November 2022. The data was collected from six health facilities in Kiambu County.

Inclusion criteria - HCWs who had direct interactions with neonates or were involved in providing or handling CHX cord antiseptic during the study period, and who had at least 6 months of experience with cord care.

Exclusion criteria - HCWs who were not directly involved in newborn care or who declined to provide informed consent and who had less than 6 months of experience with cord care.

Data collection

All interviews were conducted by two members of the study team (JMG and BNO) in English, but the participants were allowed to respond in English or Kiswahili. With the help of a translator (BNO) who identified as a male, the IDIs were audio-recorded and transcribed the interviews verbatim directly from Kiswahili (where applicable) into English. JMG and BNO conducted 38 IDIs were conducted between 6th October 2022 to 9th November 2022.

Upon arrival at the various study sites, verbal consent was initially sought from the manager of each facility unit, followed by written consent from the hospital medical superintendent. Providers were purposefully selected based on their interaction with neonates or CHX cord antiseptic products, their availability, and their willingness to have the interviews recorded.

Data collection was managed using Kobo Toolbox software, and interviews were conducted in English, following a semi-structured questionnaire.

Data Management and Analysis

Kobo Toolbox software was used to collect and manage data, with all data coded to conceal the participants' identities. Interview transcripts were imported into NVivo software to assist in the classification, sorting, and coding of data into central themes and subthemes. The coding process followed a two-cycle approach. In the first cycle, value coding was used to capture participants' attitudes and values, reflecting their perceptions and experiences. The second cycle involved focused and pattern coding. Focused coding developed specific categories without focusing on their properties, while pattern coding grouped similar codes into meaningful units for theme identification [23–25].

NVivo's platform played a crucial role in facilitating this multi-level coding process, ensuring consistency in the analysis framework by identifying recurring patterns and connections. The research questions and interview guide informed the coding frame, allowing for accurate classification of notable data segments to reveal dominant themes. The integration of value, focused, and pattern coding in both cycles ensured a comprehensive and structured analysis of the interview data [23–25].

Ethical approval

Approval was obtained at various administrative levels, including county and sub-county authorities, before the study commenced. Ethical clearance was secured from the University of Nairobi/Kenyatta National Hospital (KNH) Ethical Review Committee (ERC) [P470/05/2022], County Government of Kiambu Department of Health Services, Kiambu County [KIAMBU/HRDU/22/09/14/RA_GITHINJI], and a research license obtained from the National Commission for Science, Technology and Innovation [NACOSTI/P/22/20446]. The participating facilities also provided permission to conduct the study in their facilities.

Prior to the initiation of data collection, participants were informed about the study's objectives and their role as respondents, including the importance of their continued involvement until the study's conclusion. This was conducted by JMG and BON. They were assured that participation was entirely voluntary and that they had the right to withdraw at any time. The researcher explained that all data and discussions would be recorded solely for the purposes of the study, and consent for this was obtained through the signing of a consent form. All data collected by the researcher was treated as confidential and utilized exclusively for this study. Access to the materials was restricted to the investigators, and stringent data management protocols were implemented to uphold the confidentiality of the study subjects.

Results

Demographic characteristics of participants

The mean age of the 38 health workers was 35 years, with the lowest age recorded as 23 and the highest age recorded as 57 years.

At the time of the study, the county had a staff shortage, and those found on duty were all interviewed, totaling 38 healthcare providers. Among them, nurses comprised the largest group at 50.0% (n = 19), followed by pediatricians at 15.7% (n = 6), pharmacists and pharmaceutical technologists both at 10.5% (n = 4 each), medical officers at 7.8% (n = 3), and clinical officers at 5.2% (n = 2), all working in ANC, Maternity, and/or PNC.

The participants had an average practice duration of 9 years, ranging from a minimum of 6 months to a maximum of 30 years.

Findings

The transcripts drawn from recordings of in-depth interviews held with selected health workers were analyzed, and themes emerged as 33 files with 52 references. Some themes mirrored the guiding questions in the semi-structured questionnaire.

Sub-themes with high references were surgical spirit, raising awareness, effectiveness, easy access, availability, ease of use, and delayed healing. Existing cord care practices were the most discussed theme among those with the fewest sub-themes, as shown in Table 1.

Table 1. The clustered analysis of transcripts from interview audio recordings. The "References" column indicates the total number of individual references that mention that specific theme within the files.

Section	Themes	References
**Existing cord care practices**		
	Surgical spirit	24
	CHX	23
	Dry Cord Care	8
	Traditional methods	4
**Advantages of CHX**		
	Effectiveness	31
	Quick healing	18
	Easy to use	13
**Barriers against provision of CHX**		
	Easy access	23
	Affordability	9
**Challenges experienced with CHX**		
	Availability	22
	Delayed healing	13
	Knowledge gap	12
	Cost	10
	Resistance	6
	Reported infections	3
**Recommendations to increase CHX use**		
	Raising awareness	44

	Increasing availability	25
	Making it affordable	8
**Use of IEC materials**	Inadequate, unavailable	52

Existing cord care practices

We sought to understand the general cord care practices in various health facilities across Kiambu County by interviewing healthcare workers (HCWs). According to Ministry of Health (MOH) guidelines, the use of chlorhexidine (CHX) for cord care has been officially recommended, with the directive being disseminated from the central government to the county level. This was confirmed by Participant 25 from Nazareth Hospital, who stated, "*first of all, we have the policy that is an order that came from Kiambu County from the Ministry of Health that chlorhexidine should be used*" (**Na_25**).

While the majority of participants acknowledged the existence of the MOH guidelines recommending CHX for cord care and confirmed that this policy had been communicated to health facilities, many reported the continued use of both surgical spirit and CHX. Notably, more HCWs mentioned that surgical spirit remained the predominant choice in practice, indicating a reluctance to fully transition from previous guidelines. In fact, 41.7% of participants reported having prior experience with surgical spirit before being required to switch to CHX, which was a relatively new product in their facilities.

A minority of participants also referred to dry cord care as part of the cord care practices in Kiambu County. Additionally, some facilities reported not using CHX at all, relying solely on surgical spirit for cord care. This variation in practice highlights the need for further investigation into the slow adoption of CHX in these settings, despite the existence of formal guidelines.

> "*And what I've noted, there is less uptake of chlorhexidine use for cord care compared to the surgical spirit. ... the pediatrician and also other practitioners [are] persistent on the use of surgical spirit as opposed to chlorhexidine.*" **G_19**

> "*So currently, in Kiambu County, there are different things that are being done. One, there' are some places and institutions that are using chlorhexidine for cord care. Then a number of institutions are using surgical spirit. There are some people who even believe on doing nothing to cord care. There are people who are using normal saline for cord care.*" **Th_37**

> *"So after birth, when you cut the cord, immediately we use the chlorhexidine, and after, we use it once a day, once a day after delivery until now the mothers goes home and you instruct them to use it until the cord dries off and falls off."* **Na_27**

> *"So once the cord has been cut and the umbilical clamp has been placed, the next thing is to maintain hygiene of the cord, and this is done by applying an antiseptic. Commonly we use chlorhexidine, either the gel or the solution. But other centers may use surgical spirit. Other centers may also use air to let it air dry naturally. So those are the methods that I'm aware that take care of the cord of the baby."* **K_36**

> *"…I just know most of the time, they don't even use that chlorhexidine. They use surgical spirit."* **Ki_18**

Despite clear directives, one of the centers in our study actively deviated from the National Guidelines, opting instead to follow its own facility-based protocols. Notably, dry cord care was most prevalent in this Level 5 facility, where the use of surgical spirit or CHX was rarely practiced by mothers and was not routinely recommended by the healthcare workers.

> *"... The guys from the Afya House when they come, they say, "No, you must use [CHX]. You must use [CHX]." But then we are agreed for us, we don't use it here, we have not been using it, and we have not had any cases of neonatal sepsis."* **Th_13**

> "*So the current cord care practice in Thika Level 5 I would say, predominantly because that's where I practice is dry cord care. I hardly do surgical spirit or chlorhexidine or any other medication directly with the cord."* **Th_12**

> *"Here in our health facility... We discussed with our pediatrician and we just decided to leave the cord just like that. Don't put anything, don't put water, don't put the spirit, and don't put chlorhexidine, as long as you keep it clean. And we have not had any case of sepsis."* **Th_13**

A minor theme emerged in which some healthcare workers (HCWs) reported that mothers used multiple cord care methods, often influenced by their past experiences and family advice. For example, if surgical spirit had been ineffective with their first baby, they would switch to CHX for subsequent children, and vice versa. In some cases, mothers who delivered during the era of previous guidelines recommending surgical spirit continued to favour it. Other practices included the use of saliva, a traditional method still common in our context, particularly among traditional mothers.

"*There is the use of … saliva, especially for the traditional mothers"* **R_5**

"*One challenge is that mothers out there, they have other methods of doing cord care. When you teach them how to do it, they go at home and they're taught by other relatives how to do it. So you find them mixing with other things. Like they will buy spirit because the other baby they used spirit, or they will use another thing like savlon that's hindering proper use of the chlorhexidine."* **Na_23**

"*some mothers are resistant to change, especially those who delivered much earlier and chlorhexidine was not there before. So if they used spirit, they still want to use spirit or maybe some other techniques that they used to use… Like using saliva or cow dung."* **Na_27**

"The common challenge that we face is that mothers believe in the use of some traditional methods like use of saliva." **R_6**

Advantages of CHX

The preferences that healthcare workers (HCWs) had for CHX in cord care were varied, largely shaped by their clinical and anecdotal experiences with different methods. Approximately half of the HCWs who expressed satisfaction with CHX did so primarily due to its perceived effectiveness in preventing omphalitis and neonatal sepsis (NNS), which reduced the need for additional healthcare visits for both mothers and newborns. Many HCWs also highlighted that CHX promoted quicker stump healing compared to other methods, provided that mothers followed proper instructions, maintained good hygiene, and began cord care immediately or shortly after

birth. Additionally, CHX was praised for being easy to use, contributing to its widespread adoption and positive outcomes.

> *"So far, the outcomes have been good. We have had limited cases of sepsis after the use of chlorhexidine."* **K_22**
>
> *"We have at least few mothers who come with sepsis afterwards, but when you ask them, maybe the hygiene was not good. But those who practice, okay, cord care with chlorhexidine as they are instructed, the outcome is good."* **Ki_16**
>
> *"The use of chlorhexidine for cord care has made work easier in Kiambu since it's easy to use and its availability, its efficacy being high has made it easier for us to manage the newborns and their mothers are even happy with the healing process using chlorhexidine gel."* **K_34**
>
> *"The effectiveness of cord care is better compared with other methods, like the surgical spirit or saliva for the traditional mothers because with the report of the mothers, it easier for them to use the cord care other than washing or cleaning the cord with the surgical spirit. Number two, in comparison with the mothers that have been using the surgical spirit and the cord care, during the postnatal checkup one week post-delivery, the mothers who have been using the cord care, the cords are healed faster compared with other methods."* **R_5**

Barriers against the provision of CHX

One of the most significant barriers to the adoption and provision of CHX for cord care was its inconsistent availability in government health facilities, even in centers where it was the recommended practice. This challenge of access was twofold: not only was there a shortage of

government-supplied CHX to health facilities, but when it was available, its cost often priced consumers out of the market, pushing them to seek more affordable alternatives.

> *"...And then the erratic supplies for the county, particularly because of those financial constraints the counties have been having have made it easy for us to try out other things because in the absence of chlorhexidine, what else was being done to allow cords to heal... the supplies and the county, it's quite irregular. The stock-outs are very, very, very common because of the budgetary constraints in the county."* **Th_12**

> *"The first challenge is limited resources from the county. That is, when receiving our delivery from KEMSA, we might also be able to receive chlorhexidine. Another challenge is from the patients. Once the drug is prescribed, some patients may not be able to purchase from the pharmacies outside."* **Ki_24**

Challenges encountered with the use CHX itself

The majority of participants who routinely used CHX in their facilities reported encountering challenges in providing it to patients. They shared diverse second-hand experiences from patients, noting that CHX was often not available in the facility in which they worked, which hindered its expeditious application on the cord after delivery.

> *"The challenges of provision of chlorhexidine gel, first of all, I'd say it's not readily available. As in, even if I sent a mother outside here to buy, I don't think it's as available as there rest."* **G_33**

In some cases, HCWs associated the use of CHX with delayed cord healing, sepsis, and prolonged cord detachment, with some mothers returning to the hospital due to infections. There were concerns over the different formulations of CHX, which led to confusion among HCWs, particularly regarding its similarity to tetracycline eye ointment and the challenges of application. Some HCWs also believed that CHX, particularly in its drop formulation, contributed to sepsis and soft tissue burns. As one healthcare worker noted:

> "*…The [CHX] drops, the mothers are coming back with septic cords. I don't know whether it's the hygiene at home or what. I don't get. But when we get a mother that comes and then we take the history, they say they bought the drops. But with the gel, no, not much sepsis. But with the drops, there is."* **K_35**

> *"Now, the problem with the gel was that either it was too thick and it kept leaving the cord moist, and so it would take longer for the cord to drop off. And so with that challenge, and of course every mother wants their cord to drop within a few weeks of administration of whatever it is they're using for cord care. So a lot of children would come with delayed cord dropping."* **Th_12**

> *"Initially, we used to use the chlorhexidine gel, which was a bit difficult to use because it required one to wash their hands or change their gloves, apply the gel on their finger, and then spread the gel on the cord and surrounding skin. The gel could also be confused with tetracycline eye ointment because of the kind of packaging it came in."* **K_36**

The data revealed a significant knowledge gap among HCWs and mothers regarding the use of CHX for cord care, with some indicating that it should be applied once a day but others

recommending it twice a day. HCWs often demonstrated preferences for alternative practices, reflecting reluctance towards CHX. These gaps were primarily due to insufficient training on CHX application, leading to a lack of awareness among mothers about proper usage. A key theme that emerged was HCWs' perception of CHX effectiveness, often shaped by feedback from mothers. These second-hand reports reinforced the view that CHX was less effective for cord care compared to other methods, influencing its provision in healthcare facilities.

> **“***What can lead into an increased use of chlorhexidine gel now that we can see it's working is that the mothers need an education. There are mothers who will not want to use chlorhexidine gel because of mythical thoughts or superstition, or that's not what they were taught by their mothers to use. So sensitization is needed. And also, sensitization among the users, those are the mothers, is needed. And also, we need to sensitize the healthcare workers because sometimes we have found mothers who have not used chlorhexidine gel because the physician who was working with the mother did not believe in the working of chlorhexidine gel. So I think even the healthcare workers need also to be told or to be taught, to be sensitized about chlorhexidine gel.”* **K_34**

Among HCWs, the knowledge gap regarding CHX use is further complicated by resistance to switch from surgical spirit, which many have successfully used in the past. This resistance is often rooted in personal experiences with both products. HCWs tend to favor surgical spirit, largely due to their familiarity with it and the perceived shortcomings of CHX.

> “*I think the fact that most of the healthcare workers were used to surgical spirit, the switch to chlorhexidine and the fact that it takes time, the cord takes time to heal, I think most people are still reluctant to start mothers on the gel. They'll prefer surgical spirit.”* **Ki_15**

The cost of CHX was also considered prohibitive for many mothers at the facility, leading HCWs to recommend more affordable alternatives. Participants expressed concerns about CHX being more expensive compared to other cord care options, which hindered its widespread adoption. Some HCWs noted that the higher cost of CHX posed a barrier to access, causing mothers to opt for cheaper alternatives like surgical spirit. Additionally, some HCWs perceived that, despite its higher cost, CHX was not as effective as other options, contributing to delayed cord healing and infection in certain cases.

> *"Secondly, the cost. It's a challenge because the mothers are not able to afford it."* **G_33**

Recommendations to increase CHX use

HCWs consistently emphasized several key recommendations to improve the adoption and effectiveness of CHX in cord care. They highlighted the need for greater awareness and education among both HCWs and patients on the benefits and proper use of CHX, as knowledge gaps were still prevalent. Many HCWs stressed the importance of making CHX more accessible, either by including it in maternity packages or ensuring its availability in hospital pharmacies to facilitate timely application immediately after delivery, which is critical for its efficacy. Affordability was another recurring theme, with HCWs recommending that CHX be priced more affordably and possibly covered by insurance schemes such as NHIF or *Linda Mama* to ease the financial burden on mothers. Additionally, they suggested that comprehensive training programs be implemented for midwives, doctors, and pediatricians to increase their understanding of CHX and its benefits, which would, in turn, allow them to better educate and support patients in adopting this cord care

method. These improvements, they believed, would help overcome current barriers and lead to wider uptake of CHX in cord care practices.

> *“Yes. So number one, it's the cost. It is readily available to them considering that it is covered by NHIF, number one. So the matter of the cost is out of the question..”* **Na_27**

> *“Suggest that the hospital should source for more of the drug to ensure that it's constantly available for use to increase its uptake.*
>
> *Interviewer: If it was available, do you think everybody would use.*
>
> *Interviewee: Yeah.”* **K_22**
>
> *“I think we need proper training for the staff, especially in maternity, even for us working in the postnatal clinics, because I understand the gel should be started immediately after birth. So maybe that might be one of the contributing factors as to why the cord does not... It takes time to heal.”* **Ki_15**
>
> *“The other one is educating, first of all, the healthcare worker on how to use it so that the healthcare worker is empowered to also educate the mothers on how to use it.”* **Th_11**

Opinions on Information, Education, and Communication (IEC) materials

IEC materials depicting the application of CHX were found to be largely unavailable and inadequate in healthcare facilities. A significant majority of respondents (86.1%) reported that these materials were not readily accessible in most hospital units, resulting in ineffective promotion of CHX utilization. Participants reached a consensus on the absence of educational resources in the wards where they worked. In cases where materials did exist, they were often not suitable for illiterate women, who frequently resorted to traditional methods of cord care.

To address this gap, participants suggested developing more inclusive IEC materials, such as pictorials and patient-friendly infographics, to ensure that all mothers could benefit from the health education provided. HCWs recommended tailoring resources specifically for their needs, including physical charts as reminders at workstations, along with Continuous Medical Education (CME) sessions and training programs. For patients, the proposed materials included charts, pictograms, and illustrations using accessible language. Additionally, these patient-friendly IEC materials should be strategically placed in locations such as antenatal clinics and labor wards, so that mothers are informed about the importance of CHX and can prepare to obtain it before delivery. Observations made by the participants included the following:

> *"But in the facility in Githunguri, I've not seen any [IECs]. And I think CME is about the chlorhexidine would really help in creating awareness of the... Is it the drug? Yeah.everywhere, in every ward, in the postnatal rooms, and in labor ward, and also here in antenatal."* **G_29**

> *"On the issue of IEC materials, they're not available in our facility. If they can be effective and brought down to the facilities, I would recommend they be more pictorial. By a glance, someone is able to know what it is and how it can be used and the readings big enough to be legible. Yes."* **G_33**

> *"The IEC material would help the staff here be at least more conversant for* those who are not conversant with chlorhexidine gel, its use, its application, the period which the patient is supposed to use. And through that, most staff will be able... Through the information the staff acquire, they'll be able to make more informed decisions of even escalating the matter to facilitate the increase of the supply of the chlorhexidine gel in the facility and also acts

a refresher for them whereby it leads to a wider knowledge and understanding of its benefits as opposed to other previous methods that were used of cord care…The IECs are not available at the facility at the moment as to my knowledge." **K_22**

"For the charts, they should be bigger, and they should provide more charts so that we can put maybe in the wards and to educate the mothers. And for those mothers who are illiterate who cannot read, the wordings are many than the pictures. The pictures are better illustrating of how to use the chlorhexidine." **Na_27**

Discussion

This study aimed to investigate the experiences of HCWs in Kiambu County regarding newborn cord care practices. The study revealed that the majority of HCWs favored the use of surgical or methylated spirit for cord care, similar to the findings of previous research involving mothers in Nigeria [9], which indicated that 84.25% of mothers, particularly those with secondary and tertiary education, employed methylated spirits. This was against the recommended MOH practices for cord care in Kenya. However, in our study, the adoption of CHX is increasing. Notably, 41.7% of the HCWs reported transitioning from surgical spirit to CHX following its introduction in their facilities. The continued use of surgical spirit and dry cord care practices suggests that the adoption of CHX among mothers with infants in Kiambu County has not yet been fully embraced.

The participants in this study reported diverse experiences with the use of CHX for cord care. Key concerns included the cost of CHX products, delayed healing, and varying perceptions of its effectiveness. While some HCWs noted CHX's ease of use, effectiveness in promoting quick healing, and reduction of sepsis, others cited concerns regarding delayed healing and affordability.

The cost of CHX, which was quoted between Ksh. 120 to 200 (USD 0.93 to 1.55), was perceived as expensive by some clients, particularly when compared to more affordable alternatives such as methylated spirit (Ksh. 50, USD 0.34) or dry cord care, which incurred no cost. This contrasts with the findings of Opanga et al., who reported that CHX gel was available at less than Ksh 40 in private facilities and pharmacies, and was free in public hospitals, yet with only a minimal uptake of 1.3% [5]. In another study conducted in rural Bungoma County, Kenya, Muriuki et al. found that CHX was provided free of charge to mothers after delivery, but noted that improper use and misunderstandings regarding its application negatively impacted uptake, along with concerns about delayed cord separation [8]. Despite these challenges, positive feedback from participants, particularly around the ease of use, prevention of sepsis, and faster cord separation, suggests potential benefits. A participant in Opanga et al.'s (2022) study reported early cord separation, with the cord drying and detaching after just three days, highlighting the promise of CHX in improving neonatal outcomes when used appropriately.

The majority of HCWs interviewed indicated that they encountered various challenges in the provision of CHX, with the most commonly reported issues being availability and cost. The emphasis on accessibility and cost is particularly noteworthy, as the inclusion of CHX in the Kenya Essential Medicines List was intended to enhance its availability in the public sector, and this highlights a gap in enforcement of this directive in our context [26]. The overarching goal of healthcare systems is to ensure that all patients have consistent access to a comprehensive range of suitable, high-quality, and affordable essential medicines, as emphasized by the World Health Organization. Essential medicines, including CHX, are critical components of healthcare, recognized alongside clean water, safe food supply, and adequate housing as fundamental necessities; their absence constitutes a failure to uphold universal human rights [27,28]. Therefore,

addressing the barriers to the effective provision of CHX is essential to improving neonatal care and advancing public health objectives.

Medicines, including essential items, frequently experience stock-outs in developing countries [27,28]. In Kenya, where health services are devolved (the national government “shares” government functions with smaller county governments), the impact of these stock-outs varies across the different counties. To mitigate the occurrence of stock-outs for essential medications on the package list, it is crucial for public sector facilities to ensure timely payments to the Kenya Medical Supplies Authority for their orders, a problem that has plagued the Kenyan medical supply chain [29,30]. Conversely, non-public facilities report infrequent stock-outs due to differing and strengthened procurement processes. Stock-outs were identified as a significant concern by Ambale et al. (2020) at Kangundo Hospital [31]. Consequently, participants reported a lack of sustained experience with the products, which undermined their confidence in persuading mothers to purchase them upon leaving the healthcare facilities. Furthermore, to achieve optimal coverage, particularly for economically disadvantaged families, subsidization may be necessary [32].

HCWs reported inadequate training in CHX cord care, leading to significant knowledge gaps and unfamiliarity with recommended guidelines. This resulted in variations in the reported frequency of CHX application. This finding aligns with Ambale et al. (2020), who linked the lack of training and clear instructions to the ineffectiveness of CHX gel [31]. To address research-driven shifts, standardized guidelines for CHX use are essential. Ongoing medical education for all cord care personnel is crucial. Participants also emphasized the need for increased media publicity to enhance consumer knowledge, in line with the sensitization efforts recommended by Opanga et al. [5].

According to the Kenya Demographic and Health Survey (KDHS), 89.2% of births in Kiambu County occurred in hospitals, with 98% of these deliveries attended by medical professionals [33]. This rate is notably higher than the 54% and 64% reported in Pemba (Tanzania) and Zambia, respectively [15,16]. However, having a medically assisted delivery does not necessarily guarantee a reduced risk of infection, as many medical centers in our context fail to meet minimal infection prevention standards [34]. Furthermore, potentially hazardous procedures that raise concerns in home delivery settings are often performed on women and infants before they are discharged within hours of birth (Countries Moving Forward, 2014). This underscores the justification for the use of CHX in all settings as recommended by the WHO.

Most HCWs in our study suggested several strategies to enhance the uptake of CHX, including increasing awareness through media campaigns, improving the availability and affordability of the product, and fostering adaptability among healthcare providers. Participants emphasized the importance of recommending CHX to mothers and providing education on its proper use. These sentiments are echoed in the existing literature as this resistance to CHX has been previously described in similar settings [8,36–38].

Both the quantity and quality of IEC materials on CHX need improvement. Visual aids and illustrations should be prioritized over text to enhance mothers' understanding of proper CHX use. IEC is crucial for ensuring correct usage, infection control, patient safety, and adherence to best practices. The study found that while most health facilities had some IEC materials on CHX, these resources were limited and lacked engaging illustrations to support learning. This finding aligns with those of the Bungoma County study [8] and further highlights a pressing need to improve these materials for HCWs.

CHX implementation should be tailored to each county's needs, following WHO guidelines, to effectively prevent neonatal infections like omphalitis. Health education is key to successful rollout, ensuring mothers receive accessible information on its use. Additionally, healthcare facilities must improve CHX availability and accessibility to enhance its adoption and effectiveness.

Conclusion

In Kiambu County, less than half of the healthcare workers delivering services preferred the use of CHX for cord care to prevent omphalitis. The perspectives among healthcare providers regarding the use of CHX are mixed; while some recognize its effectiveness, ease of use, and affordability, others perceive it as expensive and unnecessary. Furthermore, one healthcare facility actively practiced cord care that was against the National Guidelines of the country. Several factors influence the provision of CHX, including affordability, accessibility and perceived effectiveness. This disparity in perceptions and the varied factors impacting CHX use underscore the need for targeted education and resources to foster a unified approach to neonatal care. Addressing these issues will be pivotal in enhancing the overall health outcomes for newborns in the region.

References

[1] Clarke-Pounder JP, Golden WC. Omphalitis. The 5-Minute Pediatric Consult, 8th Edition 2022:656–7. https://doi.org/10.1097/pec.0000000000002918.

[2] López-Medina MD, Linares-Abad M, López-Araque AB, López-Medina IM. Dry care versus chlorhexidine cord care for prevention of omphalitis. Systematic review with meta-analysis. Rev Lat Am Enfermagem 2019;27. https://doi.org/10.1590/1518-8345.2695.3106.

[3] Clarke-Pounder JP, Golden WC. Omphalitis. The 5-Minute Pediatric Consult, 8th Edition 2022:656–7. https://doi.org/10.1097/pec.0000000000002918.

[4] Quattrin R, Iacobucci K, De Tina AL, Gallina L, Pittini C, Brusaferro S. 70% Alcohol Versus Dry Cord Care in the Umbilical Cord Care: A Case-Control Study in Italy. Medicine 2016;95. https://doi.org/10.1097/MD.0000000000003207.

[5] Opanga Y, Karanja S, Abdullahi Z, Gichuki R, Ahmed A, Tupeiya V, et al. Enablers and Barriers to Chlorhexidine Use in Umbilical Cord Care: Voices of Care Givers and Healthcare Providers in Selected Counties in Kenya. East African Journal of Health and Science 2022;5:72–86. https://doi.org/10.37284/EAJHS.5.2.995.

[6] Afolaranmi TO, Hassan ZI, Akinyemi OO, Sule SS, Malete MU, Choji CP, et al. Cord Care Practices: A Perspective of Contemporary African Setting. Front Public Health 2018;6:309563. https://doi.org/10.3389/FPUBH.2018.00010/BIBTEX.

[7] Karumbi J, Mulaku M, Aluvaala J, English M, Opiyo N. Topical umbilical cord care for prevention of infection and neonatal mortality. Pediatr Infect Dis J 2013;32:78–83. https://doi.org/10.1097/INF.0B013E3182783DC3.

[8] Muriuki A, Obare F, Ayieko B, Matanda D, Sisimwo K, Mdawida B. Health care providers' perspectives regarding the use of chlorhexidine gel for cord care in neonates in rural Kenya: implications for scale-up. BMC Health Serv Res 2017;17. https://doi.org/10.1186/S12913-017-2262-8.

[9] Asiegbu U V., Asiegbu OG, Ezeonu CT, Ezeanosike OB, Onyire BN, Asiegbu U V., et al. Determinants of Cord Care Practices among Mothers in Abakaliki, Ebonyi State, South East, Nigeria. Open J Prev Med 2019;9:43–50. https://doi.org/10.4236/OJPM.2019.95005.

[10] Geneva: World Health Organization. WHO recommendations on maternal and newborn care for a positive postnatal experience. 2022.

[11] Imdad A, Bautista RMM, Senen KAA, Uy ME V., Mantaring JB, Bhutta ZA. Umbilical cord antiseptics for preventing sepsis and death among newborns. Cochrane Database of Systematic Reviews 2013;2013. https://doi.org/10.1002/14651858.CD008635.pub2.

[12] Kenya M of PH and S, Kenya M of MS. National Guidelines for Quality Obstetrics and Perinatal Care. 2009.

[13] Kenya: Ministry of Health. Basic Paediatric Protocols. 2022.

[14] Mullany LC, Arifeen S El, Khatry SK, Katz J, Shah R, Baqui AH, et al. Impact of Chlorhexidine Cord Cleansing on Mortality, Omphalitis, and Cord Separation Time Among Facility-Born Babies in Nepal and Bangladesh. Pediatr Infect Dis J 2017;36:1011. https://doi.org/10.1097/INF.0000000000001617.

[15] Semrau KEA, Herlihy J, Grogan C, Musokotwane K, Yeboah-Antwi K, Mbewe R, et al. Effectiveness of 4% chlorhexidine umbilical cord care on neonatal mortality in Southern Province, Zambia (ZamCAT): a cluster-randomised controlled trial. Lancet Glob Health 2016;4:e827–36. https://doi.org/10.1016/S2214-109X(16)30215-7.

[16] Sazawal S, Dhingra U, Ali SM, Dutta A, Deb S, Ame SM, et al. Efficacy of chlorhexidine application to umbilical cord on neonatal mortality in Pemba, Tanzania: a community-based randomised controlled trial. Lancet Glob Health 2016;4:e837–44. https://doi.org/10.1016/S2214-109X(16)30223-6.
[17] Shwe DD, Afolaranmi TO, Egbodo CO, Musa J, Oguche S, Bode-Thomas F. Methylated spirit versus chlorhexidine gel: A randomized non-inferiority trial for prevention of neonatal umbilical cord infection in Jos, North-Central Nigeria. Niger J Clin Pract 2021;24:762–9. https://doi.org/10.4103/NJCP.NJCP_535_20.
[18] Ministry of Health; Kenya. The Kenya Guidelines for the Use of Chlorhexidine Digluconate 7.1% for Prevention of Newborn Umbilical Cord Infection. 2022.
[19] Githinji JM, Chepchirchir A, Juttla PK, Nduati R. Adoption and factors associated with 7.1% chlorhexidine digluconate cord care standards in public health facilities in Kiambu County, Kenya. Clin Epidemiol Glob Health 2024;29:101781. https://doi.org/10.1016/J.CEGH.2024.101781.
[20] Juttla PK, Ndiritu M, Milliano F, Odongo AO, Mwancha-Kwasa M. Knowledge, attitudes and practices towards COVID-19 among healthcare workers: A cross-sectional survey from Kiambu County, Kenya. PLoS One 2024;19:e0297335. https://doi.org/10.1371/journal.pone.0297335.
[21] Mwancha-Kwasa M, Admani R, Mbuga M, Maina M, Mwangi J, Ng'ang'a L, et al. Comparing labour induction outcomes using misoprostol and dinoprostone in term pregnancies: A retrospective study at Kiambu Level 5 Hospital between 2018 and 2020. PLoS One 2024;19:e0304631. https://doi.org/10.1371/journal.pone.0304631.
[22] Rahimi S, khatooni M. Saturation in qualitative research: An evolutionary concept analysis. Int J Nurs Stud Adv 2024;6:100174. https://doi.org/10.1016/J.IJNSA.2024.100174.
[23] Saldaña J. Goodall's Verbal Exchange Coding. Http://DxDoiOrg/101177/1077800415603395 2015;22:36–9. https://doi.org/10.1177/1077800415603395.
[24] Stuckey H. The second step in data analysis: Coding qualitative research data. Journal of Social Health and Diabetes 2015;03:007–10. https://doi.org/10.4103/2321-0656.140875.
[25] Salmona M, Kaczynski D. Don't Blame the Software: Using Qualitative Data Analysis Software Successfully in Doctoral Research. Forum Qualitative Sozialforschung / Forum: Qualitative Social Research 2016;17. https://doi.org/10.17169/FQS-17.3.2505.
[26] Ministry of Health. Kenya Essential Medicines List 2019. Nairobi: 2019.
[27] Perehudoff K. Universal access to essential medicines as part of the right to health: a cross-national comparison of national laws, medicines policies, and health system indicators. Glob Health Action 2020;13:1699342. https://doi.org/10.1080/16549716.2019.1699342.
[28] Brhlikova P, Persaud N, Osorio-De-Castro CGS, Pollock AM. Essential medicines lists are for high income countries too. BMJ 2023. https://doi.org/10.1136/bmj-2023-076783.
[29] Kariuki J, Njeru MK, Wamae W, Mackintosh M. African Centre for Technology Studies Local Supply Chains for Medicines and A Working Paper from the Industrial Productivity, Health Sector Performance and Policy Synergies for Inclusive Growth (IPHSP) research project. n.d.

[30] Health Action International. Prices and Availability of Locally Produced and Imported Medicines in Kenya 2018. https://haiweb.org/media-resource/prices-and-availability-of-locally-produced-and-imported-medicines-in-kenya/ (accessed October 6, 2024).
[31] Ambale C, Ngatia B, Nthusi J, Mukonzo J. Assessment of the Use Of Chlorhexidine Digluconate Gel for Cord Care at Kangundo Level 4 Hospital. International Journal of Scientific and Research Publications (IJSRP) 2020;11:308–15. https://doi.org/10.29322/ijsrp.11.01.2021.p10932.
[32] Coffey PS, Metzler M, Islam Z, Koehlmoos TP. Willingness to pay for a 4% chlorhexidine (7.1% chlorhexidine digluconate) product for umbilical cord care in rural Bangladesh: a contingency valuation study. BMC Int Health Hum Rights 2013;13. https://doi.org/10.1186/1472-698X-13-44.
[33] National Bureau of Statistics Nairobi K. Kenya Demographic and Health Survey 2022 Key Indicators Report. 2023.
[34] Vilar-Compte D, Camacho-Ortiz A, Ponce-de-León S. Infection Control in Limited Resources Countries: Challenges and Priorities. Curr Infect Dis Rep 2017;19:1–7. https://doi.org/10.1007/S11908-017-0572-Y/TABLES/1.
[35] Cord care with 7.1% chlorhexidine digluconate saves newborn lives Chlorhexidine for Umbilical Cord Care: A new, low-cost intervention to reduce newborn mortality 2.95 million. 2014.
[36] Ans M, Hussain M, Ahmed F, Khan KJ, Abbas S, Sultan M, et al. Umbilical Cord Care Practices and Cord Care Education of Mothers Attending Health Care (Pakistan Prospect). Health N Hav 2023;15:20–32. https://doi.org/10.4236/HEALTH.2023.151002.
[37] Tadesse Y, Mussema Abdella Y, Tadesse Y, Mathewos B, Kumar S, Teferi E, et al. INTEGRATING CHLORHEXIDINE FOR CORD CARE INTO COMMUNITY BASED NEWBORN CARE IN ETHIOPIA. 2019.
[38] Coffey PS, Brown SC. Umbilical cord-care practices in low- and middle-income countries: A systematic review. BMC Pregnancy Childbirth 2017;17:1. https://doi.org/10.1186/S12884-017-1250-7/TABLES/3.

Supporting information

S1 File. Raw data. This document contains the transcriptions of the IDIs.

Click here to access/download
Supporting Information
S1 CHX.pdf
