# Supplementary material for: “...In Kiambu county, there are different things being done”: A qualitative exploration of healthcare workers’ experiences with cord care practices
Source: PLoS One. 2025 Jun 18;20(6):e0326506. doi: 10.1371/journal.pone.0326506 (PMC12176292; doi:10.1371/journal.pone.0326506)
Supplement: Attachment — Submitted filename: Comments_GB.docx [file pone.0326506.s002.docx]

This manuscript explores an important topic. Cord care practices and CHX implementation in a Kenyan county. While the study is timely and relevant, several critical areas require improvement to strengthen its scientific contribution. Detailed, section-wise suggestions are given below

**Background:**

- Add local epidemiological data: estimates of NNS, omphalitis, or mortality attributable to cord infections which would assist to understand the magnitude of the problem and strengthen the rationale for the study.
- This paragraph (third) is confusing. Contradictory evidence is described but not synthesized. what is the current consensus or lack thereof? Would recommend focus more the updated recommendation of WHO as these recommendations are made from multiple and multicountry studies and based on strong evidences.
- What was the specific evidence from Bangladesh study is missing here. Would suggest to clarify the conflict for clear understanding of the readers.
- "CHX protocol” is vague. Would recommend to specify if referring to 4% CHX gel, application frequency, or WHO guidance.
- The rationale is very weak. There is no mention of the systemic barriers (supply chain, training gaps, facility-level enforcement) that might present in the low-resouce settings which would provide context for CHX underutilization. Additionally, there is no mention whether any previous studies were conducted in Kenya or in the similar settings to understand HCW perspective, any gaps and limitation of those studies and what more this study may address particularly. Would strongly suggest to address these things to make the background scientifically sounder and more comprehensible.

**Method:**

- The design is identified as “exploratory qualitative,” but the justification for this choice is missing. Explain why this design was most suitable for assessing HCWs’ experiences.
- Lacks demographic details (cadre mix, gender, years of experience, urban vs rural distribution) which is crucial for assessing diversity and representativeness.
- Mention the qualification of JMG and BNO whether they received any qualitative interview training.
- Data management is briefly mentioned, but no info on data encryption, password protection, or cloud security for Kobo/NVivo files. would suggest to address.

**Result:**

- Add a table for clear understanding summarizing:
- Number of HCWs by cadre (nurses, pediatrician, pharmacist, MO, clinical officers, etc.)
- Facility type (Level 5/4/3, public/private/faith-based)
- Urban vs rural distribution.

**Findings:**

- Merge overlapping themes/sub-themes (e.g., “availability” and “access”) and synthesize common patterns across quotes rather than listing them sequentially.
- This findings section provides a rich, grounded understanding of cord care practices and CHX implementation challenges in a Kenyan county. However, the current presentation is verbose, and could benefit from clearer synthesis and critical reflection. Consider a thematic framework diagram to visually depict intensity and co-occurrence of themes across categories (e.g., advantages, barriers). With revisions focused on thematic clarity, and contextual framing, this section could substantially enhance the manuscript’s contribution to the research in newborn health.
- While quotes are tagged by anonymized ID, more information (e.g., facility type or cadre) would help interpret responses.

**Discussion:**

- Would suggest to emphasize what new insights this study offers beyond confirming previous finding. For instance, specific systems-level insights about devolution and supply chains in Kiambu.
- The discrepancy in reported CHX pricing between studies (e.g., Ksh. 40 vs. 200) requires deeper discussion. Is this due to procurement models, subsidies, or corruption?
- While lack of training is mentioned, the scope or standard of existing training programs is not elaborated. Are they nonexistent, outdated, or misaligned with WHO protocols?

**Conclusion:**

- The conclusion blends findings and recommendations without clearly differentiating them. This weakens the take-home message.
- "One healthcare facility actively practiced cord care against the National Guidelines” is serious but underexplaine. This needs clarification or should be omitted from the conclusion unless further elaborated.
- Consider including a final sentence with a stronger policy implication (e.g., need for CHX to be prioritized in county-level budgets, routine supervision, or national monitoring frameworks)
